# Influence of Drug Properties, Formulation Composition, and Processing Parameters on the Stability and Dissolution Performance of Amorphous Solid Dispersions-Based Tablets

**DOI:** 10.3390/polym17182484

**Published:** 2025-09-14

**Authors:** Ioannis Pantazos, Maria Poimenidou, Dimitrios Kouskouridas, Evangelos Tzaferas, Vasiliki Karava, Christos Cholevas, Afroditi Kapourani, Panagiotis Barmpalexis

**Affiliations:** 1 Laboratory of Pharmaceutical Technology, Division of Pharmaceutical Technology, School of Pharmacy, Faculty of Health Sciences, Aristotle University of Thessaloniki, 54124 Thessaloniki, Greece; ipantazo@pharm.auth.gr (I.P.); mpoim@pharm.auth.gr (M.P.); kouskoud@pharm.auth.gr (D.K.); etzaferas@pharm.auth.gr (E.T.); vekarava@pharm.auth.gr (V.K.); ccholevas@pharm.auth.gr (C.C.); pbarmp@pharm.auth.gr (P.B.); 2 Natural Products Research Centre of Excellence-AUTH (NatPro-AUTH), Center for Interdisciplinary Research and Innovation (CIRI-AUTH), 57001 Thessaloniki, Greece

**Keywords:** amorphous solid dispersions, downstream process, physical stability, supersaturated dissolution performance

## Abstract

Polymeric-based amorphous solid dispersions (ASDs) represent a widely employed strategy for enhancing the oral bioavailability of poorly water-soluble drugs, but their successful implementation in solid dosage forms requires careful optimization of both formulation composition and compaction parameters. In this study, the performance of polymeric-based ASD tablets were investigated using two model active pharmaceutical ingredients (APIs) with distinct glass-forming abilities (GFAs) and physicochemical characteristics: (1) indomethacin (IND, a good glass former) and (2) carbamazepine (CBZ, a poor glass former). ASDs were prepared at various API-to-polyvinylpyrrolidone (PVP) ratios (10:90, 20:80 and 40:60 *w*/*w*) and incorporated into round-shaped tablets at different ASD loadings (20% and 50% *w*/*w*). The impact of compaction pressure and dwell time on the mechanical properties, disintegration, and supersaturation performance was assessed, both immediately after preparation and following three months of storage at 25 °C and 60% relative humidity. Solid-state analysis confirmed the amorphous state of the APIs and revealed the development of API–polymer molecular interactions. Supersaturation studies under non-sink conditions demonstrated that dissolution behavior was strongly influenced by drug loading, polymer content, and compaction conditions, with CBZ formulations exhibiting faster release but greater susceptibility to performance loss during storage. The comparative evaluation of IND and CBZ highlights the critical role of API properties in determining the physical stability and dissolution performance of ASD tablets, underscoring the need for API-specific design strategies in ASD-based formulation development.

## 1. Introduction

Oral administration remains the most prevalent and preferred route for drug delivery, primarily due to its convenience, non-invasive nature, and ability to improve patient adherence [1,2]. However, a major challenge in oral dosage form development lies in addressing the poor aqueous solubility of active pharmaceutical ingredients (APIs). It is estimated that approximately 40% of marketed drugs and up to 90% of drug candidates currently in development exhibit low water solubility, which directly impairs their bioavailability [3,4]. To address this critical challenge, a wide array of formulation strategies has been developed over the past several decades. These include particle size reduction, polymorphic modification, crystal lattice modification (such as amorphization or co-crystal formation), incorporation into carrier systems (e.g., eutectic mixtures, solid dispersions, solid solutions), complexation with surfactants or cyclodextrins, chemical modifications (e.g., salt formation, prodrug strategies), lipid-based delivery platforms, and pH-modulating systems [4,5,6].

Among these approaches, amorphous solid dispersions (ASDs) have attracted considerable attention due to their capacity to enhance a drug’s solubility and dissolution rate [7,8]. A key mechanism by which ASDs enhance the oral bioavailability of poorly soluble drugs is through the generation of a supersaturated solution upon dissolution. This elevated concentration facilitates enhanced drug permeation across the intestinal epithelium. However, the principal limitation of ASDs lies in their intrinsic thermodynamic instability. The amorphous state, being a high-energy configuration, is inherently prone to recrystallization when exposed to environmental stress such as moisture, elevated temperatures, or mechanical forces encountered during manufacturing, storage, and distribution. Recrystallization not only diminishes the supersaturation potential but also reduces the dissolution rate and, consequently, the in vivo performance of the formulation. Therefore, careful design and optimization of ASD systems are essential to mitigate these risks and ensure sustained physical stability [9].

The physical stability of ASDs is influenced by several well-established factors, including the molecular mobility of the API, the nature of drug–polymer interactions, and environmental storage conditions [10,11,12,13]. While these parameters have been extensively studied, the majority of studies have primarily focused on the stability of ASD intermediates—those formulations obtained directly after primary manufacturing processes such as hot-melt extrusion or solvent evaporation. However, for ASDs to be clinically viable or commercially available, these intermediates must be further processed into final dosage forms, most commonly tablets due to their patient-friendly administration, manufacturing efficiency, and cost-effectiveness. Despite the significant number of studies examining the stability and behavior of ASD intermediates, research focusing on the final tablet formulations derived from ASDs remains relatively scarce. This research gap becomes even more critical when considering that, despite the substantial scientific interest and numerous investigations into the development of effective ASD systems, as of early 2025, only a limited number of commercial formulations containing ASDs have received regulatory approval. A major contributing factor to this limited success is the considerable challenge associated with the downstream processing of ASDs into final dosage forms that meet the required pharmaceutical performance criteria—such as adequate tensile strength, disintegration time, dissolution profile, and physical stability [14,15].

In response to the recognition of this important research gap, scientific research in the field of ASDs has progressively shifted from studying the properties of neat ASDs toward addressing the challenges associated with their downstream processing into final dosage forms. A significant portion of the literature has concentrated on elucidating the role of formulation excipients in influencing both tablet performance and the physical stability of ASD-based systems [16,17]. For example, co-additives—introduced either intergranularly or extragranularly—have been shown to critically affect mechanical strength, dissolution behavior, and the recrystallization tendency of the API. In particular, published studies have demonstrated that the use of highly compressible grades of microcrystalline cellulose can enhance the manufacturability of ASD-based tablets and, importantly, may maximize their bioavailability benefits when administered as solid oral dosage forms [18]. Additionally, surfactants such as sodium lauryl sulfate (SLS) have been employed in ASDs to increase wettability and dispersion, thereby enhancing dissolution performance. Beyond this, SLS has also been reported to inhibit uncontrolled crystallization of the API during tablet dissolution, further contributing to improved drug solubility and release [19]. In contrast, the use of crystalline excipients such as lactose and mannitol has been associated with unfavorable effects, as their tendency to adsorb moisture at the surface can generate disordered interfacial regions. These regions increase molecular mobility and may promote phase transformations, including cocrystal dissociation [19,20]. Lubricants have similarly been shown to influence ASD stability. Hydrophobic lubricants like magnesium stearate (MgSt) promote nucleation and recrystallization, negatively impacting drug dissolution, whereas hydrophilic lubricants such as sodium stearyl fumarate (SSF) exhibit more favorable effects [21]. Coating processes have been associated with increased crystallinity in ASD tablets, likely due to thermal or moisture-induced surface crystallization during application [19,22], while ASD particle size has also been identified as a key determinant of both rapid disintegration and high mechanical strength [23].

Beyond excipient choice, unit operations such as granulation and roller compaction play a critical role in preserving ASD stability. Key process parameters—such as solvent type, drying temperature, and compaction force—have been shown to influence crystallinity and tablet properties [24,25,26]. Furthermore, evidence suggests that increasing ASD loading is associated with longer disintegration times across all tablet formulations, an effect that is more pronounced in systems containing hydrophilic polymers. Likewise, lower drug loading has been linked to prolonged disintegration times in hydrophilic polymer-based ASDs [25]. Beyond disintegration, it is also well established that drug loading exerts a critical influence on drug release from ASD systems. In this context, Dohrn et al. [27] reported that at certain high drug loadings, dissolution may be accompanied by rapid crystallization of the API at the surface, resulting in passivation of the ASD matrix by drug crystals and a subsequent loss of release. Although compression pressure and dwell time are also widely acknowledged as important [24,28,29,30], comprehensive studies on their specific effects remain limited. These variables affect tablet strength and disintegration, and excessive compression may even accelerate recrystallization.

Despite the valuable insights provided by prior studies, it becomes evident that the majority of research efforts have predominantly focused either on the role of excipients—such as fillers, binders, and lubricants—or on the processing techniques and environmental conditions applied during downstream operations. While these factors undeniably influence the physical stability and performance of ASD-based tablets, limited attention has been paid to the intrinsic properties of the API itself as a determinant of behavior during downstream processing. In particular, in a polymeric-based ASD, the interaction between the API’s properties and the mechanical or thermal stresses encountered during tablet manufacturing remains insufficiently explored. Among these properties, the glass-forming ability (GFA) of an API represents a critical yet underinvestigated parameter. Given that GFA fundamentally reflects an API’s tendency to resist recrystallization during cooling or processing [15,31], it is reasonable to hypothesize that APIs with different GFA classifications would exhibit distinct responses to compaction, dwell time, and formulation conditions while interacting differently with the ASD polymeric matrix/carrier. Furthermore, although the effect of drug loading on the release behavior of ASD-based tablets has been previously examined, available studies have not systematically focused on supersaturation dissolution testing. Such studies are particularly relevant, as they closely mimic gastrointestinal conditions and allow effective evaluation of the kinetics of drug recrystallization and precipitation [32].

However, to date, as far as we know, in the case of polymeric-based ASDs, no studies have directly investigated the extent to which the GFA classification of an API affects its physical stability, recrystallization propensity, and dissolution behavior when incorporated into a final tablet dosage form subjected to various manufacturing conditions. To address this gap, the present study investigates the influence of tablet compression parameters—specifically compaction pressure and dwell time—on the stability and performance of polymeric-based ASDs containing APIs from different GFA classes. Two model drugs were selected for comparison: carbamazepine (CBZ), a poor glass former (GFA I), and indomethacin (IND), a good glass former (GFA III) [33,34]. ASDs were prepared using polyvinylpyrrolidone (PVP) as a hydrophilic carrier polymer. PVP was selected as the polymeric matrix/carrier since it is among the most widely used polymers in the development of ASDs [35] and has been shown to enhance the dissolution rate of drugs in aqueous media compared with their crystalline counterparts while also reducing the driving force for API recrystallization following dissolution. However, PVP, being a homopolymer of the hydrophilic vinylpyrrolidone monomer, is highly hygroscopic and is known to absorb approximately 28 wt% of water at 25 °C and 75% relative humidity (RH) [36]. This pronounced moisture uptake is of particular interest in the context of polymeric-based ASD stability studies. In this vein, the present study investigated the effects of systematically varied compaction pressures (ranging from 50 to 250 MPa) and dwell times (from 5 to 60 s), alongside the influence of drug-to-polymer ratio and the proportion of ASD incorporated into the final tablet formulation. The resulting tablets were evaluated for mechanical strength, disintegration time, dissolution behavior under non-sink conditions, and physical stability both immediately after preparation and after three months of storage at 25 °C and 65% RH.

## 2. Materials and Methods

### 2.1. Materials

IND (form γ) and CBZ (form III) with purity ≥ 98.0%, evaluated by HPLC, were obtained from Acros Organics (Geel, Belgium) and used without any further processing. Polyvinylpyrrolidone (Kollidon^®^K25, PVP) was purchased by BASF (Ludwigshafen, Germany). Additionally, Avicel^®^ PH-102 (MCC), PARTECK^®^ CCS (croscarmellose sodium, CCS), magnesium stearate (MgSt), and talc (all from Merck KGaA, Darmstadt, Germany) were used. All other materials and reagents were of analytical or pharmaceutical grade and used as received.

### 2.2. Thermal Stability of Pure Materials

Thermogravimetric analysis (TGA) was employed to assess the thermal stability of the raw materials using a Shimadzu TGA-50 instrument (Shimadzu Corporation, Tokyo, Japan). Accurately weighed samples (5.0 ± 0.2 mg) were placed in alumina crucibles and placed on a high-sensitivity microbalance. The samples were then heated from ambient temperature to 300 °C at a constant rate of 10 °C/min under a nitrogen atmosphere, maintained at a flow rate of 50 mL/min. Throughout the thermal program, continuous monitoring of sample mass and temperature was performed to capture the thermal degradation profiles.

### 2.3. Preparation of the ASDs

ASDs were prepared using the quench cooling (QC) technique. Specifically, predetermined quantities of each API and PVP were combined to yield physical mixtures (PMs) with drug-to-polymer weight ratios of 10/90, 20/80, and 40/60 *w*/*w*. These PMs (approximately 3 g) were placed on appropriate aluminum pans and heated on a hot plate at 190 °C for IND and 210 °C for CBZ for approximately 5 min, while being continuously mixed with a glass rod until homogeneous molten dispersions were obtained. The molten samples were rapidly solidified by QC through immediate transfer into a freezer maintained at −30 °C, where solidification occurred without the use of additional cooling agents. The resulting ASDs were then gently ground using a mortar and pestle and sieved through ASTM sieves No. 100 (150 μm) and No. 80 (180 μm) to obtain particles within the 150–180 μm size range. Content uniformity of the resulting ASDs was further confirmed by sampling from three different positions of the ASDs and quantifying API concentration via UV-Vis spectroscopy (Pharma Spec UV-1700, Shimadzu Europa GmbH, Duisburg, Germany) at 318 nm for IND and 284 nm for CBZ.

### 2.4. Physicochemical and Thermophysical Characterization of the Prepared ASDs

#### 2.4.1. Differential Scanning Calorimetry (DSC)

DSC was employed to assess the physical state of the drug in the ASDs. The measurements were conducted using a Netzsch DSC 204 F1 Phoenix heat flux calorimeter (NETZSCH, Waldkraiburg, Germany). Accurately weighed samples (5.0 ± 0.1 mg) were hermetically sealed in standard aluminum pans and analyzed under a nitrogen purge at 70 mL/min to ensure an inert atmosphere.

For each sample, only a heating step was performed, starting from room temperature and increasing to the target temperature (200 °C for IND and 210 °C for CBZ) at a constant heating rate of 10 °C/min. Target temperatures were selected based on the respective melting points (~160 °C for IND and ~190–193 °C for CBZ), ensuring complete melting of the API while avoiding degradation [33]. This approach enabled direct assessment of the amorphization efficiency within the polymer matrix at each drug-to-polymer ratio. This approach was selected to evaluate the efficiency of API amorphization within the polymeric matrix at each drug-to-polymer ratio. The presence or absence of endothermic melting peaks in the resulting thermograms was used as an indicator of residual crystallinity. The melting temperature (Tm) was determined from the peak of the endothermic melting transition, while the glass transition temperature (Tg) was identified at the inflection point (midpoint) of the heat flow curve. The enthalpy of fusion (ΔH_f_) was calculated from the integrated area of the melting endotherm. The standard deviations associated with temperature and enthalpy measurements did not exceed ±1.0 °C and ±3.0 J/g, respectively. Instrument calibration for temperature was performed using high-purity benzophenone, indium, and tin, whereas enthalpy calibration was conducted with indium. Data acquisition and analysis were carried out using NETZSCH Proteus Thermal Analysis software (version 5.2.1, NETZSCH, Waldkraiburg, Germany). All DSC measurements were conducted within the first hour of ASD preparation, without intermediate storage.

#### 2.4.2. Powder X-Ray Diffractometry (pXRD)

pXRD patterns of all samples were acquired using a Bruker D2 Phaser diffractometer (Bruker, Billerica, MA, USA) equipped with a CuKα radiation source (λ = 1.5418 Å). Measurements were conducted under operating conditions of 30 kV and 100 mA. Samples were scanned over a 2θ range of 5° to 45° in continuous scan mode, employing a step size of 0.02° and a counting time of 0.5 s per step. The resulting diffraction data were used to assess the crystallinity and phase composition of the samples. All pXRD measurements were conducted within the first hour of ASD, without intermediate storage.

#### 2.4.3. Attenuated Total Reflectance-FTIR Spectroscopy (ATR-FTIR)

Molecular interactions between each API and PVP in the prepared ASDs were examined using ATR-FTIR spectroscopy. Spectra were recorded for the pure APIs, PMs, and ASDs across the wavenumber range of 650–4000 cm^−1^ using a Shimadzu IR-Prestige-21 FTIR spectrometer (Shimadzu Europa GmbH, Duisburg, Germany) equipped with a Golden Gate MKII single-reflection ATR accessory (Specac, Kent, Orpington, UK). The ATR system featured a heated diamond top plate and ZnSe lenses, providing a 45° angle of incidence, a penetration depth of 1.66 μm at 1000 cm^−1^, a refractive index of 2.4, and a spectral cut-off at 525 cm^−1^. After background correction, spectra were acquired by averaging 64 scans at a resolution of 4 cm^−1^ for each sample to ensure high-quality data for the analysis of potential hydrogen bonding or other drug–polymer interactions.

### 2.5. Tablet Manufacturing

For tablet manufacturing, the appropriate amounts of ASDs and co-excipients (sieved with 200 μm sieve) were mixed and directly compressed on a manually operated hydraulic press equipped with a 7 mm diameter flat-faced punch and die set. The die and punch set were pre-lubricated with a suspension of MgSt in acetone to minimize sticking during compression. Grinding of the ASDs and subsequent tableting were performed immediately after QC, without any intermediate storage, in order to minimize the risk of moisture uptake.

To investigate the influence of the API-to-polymer ratio on the performance of the final dosage forms, an initial series of tablets was prepared containing a fixed ASD content of 20% *w*/*w*, with varying API loadings of 10%, 20%, and 30% *w*/*w* within the ASD matrix (Formulation Type I). The selected drug-to-polymer ratios were limited to a maximum drug loading of 30% *w*/*w*, as higher drug fractions in ASDs are often associated with stability concerns, processability issues, and increased risk of phase separation, which can compromise the quality and performance of the final formulations [37].

Subsequently, to examine the effect of ASD content itself as a formulation variable, a second series of tablets was prepared containing 50% *w*/*w* ASD, in which the API concentration was held constant at 20% *w*/*w* (Formulation Type II). The exact composition of each formulation is presented in detail in Table 1.

To evaluate the effect of compaction parameters on the downstream performance of ASDs in tablet formulations, each tablet was prepared under three different compaction pressures (50, 150, and 250 MPa) and three distinct dwell times (5, 30, and 60 s). The selected dwell times exceed those typically applied in industrial practice (<1 s) and were intentionally chosen to evaluate extreme compression conditions, thereby enabling assessment of the full range of potential destabilizing effects on ASD-based tablets, consistent with previous studies [38,39].

It should be also acknowledged that, beyond compression parameters and API-to-polymer ratios, the type and concentration of the disintegrant can also influence the disintegration and subsequent supersaturation performance of ASD-based tablets. For example, literature evidence shows that SCC enables faster disintegration than sodium starch glycolate or crospovidone, with concentrations above 5% offering no additional benefit, indicating that a moderate level is sufficient for optimal performance [25]. In the present work, SCC was selected due to its established efficiency in promoting rapid disintegration through a swelling-driven mechanism. Moreover, excipient selection has been reported to impact not only disintegration but also the physical stability of ASD systems, as crystalline excipients like mannitol or lactose may accelerate recrystallization, whereas MCC or starch-based systems provide greater stabilization [20]. These findings highlight the importance of carefully tailoring disintegrant type and level during formulation optimization to balance mechanical strength, disintegration efficiency, and physical stability of ASD-based tablets. However, the present study was not designed to investigate the impact of excipient selection on the performance of IND- and CBZ-based ASDs. To maintain a controlled experimental framework, all tablet formulations were deliberately prepared with an identical excipient composition. This approach allowed for the isolation of the effects of the studied variables while minimizing potential confounding influences arising from differences in excipient functionality.

Finally, it should be noted that no protective coating was applied to the prepared tablets. Although film coatings have been proposed in ASD-based formulations as a strategy to mitigate moisture uptake and enhance dissolution performance [17], their impact strongly depends on the coating material and thickness, which may also influence the physical stability of the system [40]. Since the present study aimed to systematically evaluate the influence of formulation composition and compaction parameters on the stability and dissolution performance of ASD-based tablets, the introduction of a coating layer was intentionally avoided in order to eliminate this additional variable.

### 2.6. Characterization of Prepared Tablets

#### 2.6.1. pXRD Analysis of the Tablets

The physical state of the APIs in the final tablet formulations was evaluated using pXRD analysis (Bruker, Billerica, MA, USA). The analysis of the prepared tablet was performed using the same experimental conditions outlined previously (Section 2.4.2), enabling direct comparison between the ASDs and their corresponding compressed dosage forms. All pXRD measurements were performed immediately after preparation.

#### 2.6.2. Evaluation of Tensile Strength

The mechanical strength of the prepared tablets (7 mm diameter) was evaluated using a tablet hardness tester (TBH 220 TD, ERWEKA, Heusenstamm, Germany). Tensile strength (TS, expressed in MPa) was calculated according to the following equation [41,42]:

(1) $$TS=\frac{2F}{\pi hD}$$

where *F* is the breaking force (N), *D* is the tablet diameter (mm), and *h* is the tablet thickness (mm). Each test was conducted in triplicate to ensure data reproducibility. Tablet dimensions (diameter and thickness) were determined using a digital caliper (IAN 380693_2110, Parkside, Landau an der Isar, Germany).

#### 2.6.3. Disintegration Time Assessment

The disintegration time (DIS time) of the tablets was determined in acetate buffer (pH 4.5) using a USP-compliant disintegration tester (Pharma Test, Hainburg, Germany) operated at 37 ± 0.5 °C, following the procedure outlined in USP <701>. Disintegration time was defined as the interval required for the complete breakdown of the tablet, evidenced by the passage of all fragments through the mesh screen of the apparatus. Each formulation was tested in triplicate, and the mean disintegration time along with the corresponding standard deviation was calculated.

### 2.7. IND/CBZ Solubility and Dissolution Studies

#### 2.7.1. API’s Crystalline Saturation Solubility Determination

The saturation solubility of the crystalline forms of IND and CBZ was determined in acetate buffer at pH 4.5, which was also selected as the dissolution medium to ensure consistency across experiments and to enable accurate design and interpretation of the supersaturation studies. An excess amount of each API was added to 50 mL of the buffer and stirred at 37 °C for 24 h to ensure equilibrium. Following equilibration, the suspensions were filtered through a 0.45 μm polyvinylidene fluoride (PVDF) syringe filter. The concentration of dissolved API was then quantified by ultraviolet (UV) spectroscopy using a UV–Vis spectrometer (Pharma Spec UV-1700, Shimadzu Europa GmbH, Duisburg, Germany). Calibration curves were constructed in ethanol across a concentration range of 1–20 μg/mL and were found to be linear for both APIs. For IND, the calibration curve was described by the equation *y* = 0.017*x* + 0.008 with R^2^ = 0.9999, while for CBZ, the calibration curve was *y* = 0.0502*x* + 0.0137 with R^2^ = 0.9998. Absorbance measurements were performed at 318 nm for IND and 284 nm for CBZ. All measurements were conducted in triplicate. The saturation solubility in acetate buffer pH 4.5 was determined to be 0.010 ± 0.001 mg/mL for IND and 0.21 ± 0.003 mg/mL for CBZ.

To assess whether the solubility of the model drugs was influenced by the presence of PVP, identical solubility experiments were also performed in acetate buffer (pH 4.5) containing pre-dissolved PVP at a concentration of 2.7 mg/mL, corresponding to the maximum polymer concentration observed during the dissolution studies. The results demonstrated that the solubility of both IND and CBZ remained unchanged in the presence of PVP, indicating that the polymer did not alter the equilibrium solubility of the APIs under the studied conditions.

#### 2.7.2. In-Vitro Dissolution Studies Under Non-Sink Conditions

Supersaturated dissolution testing of the prepared tablet formulations was performed using a USP Apparatus II dissolution tester (PT-DT7, Pharma Test AG, Hainburg, Germany). The dissolution medium consisted of acetate buffer at pH 4.5, maintained at 37 ± 0.5 °C, with a paddle rotation speed set to 100 rpm. To mimic the finite-volume environment of the gastrointestinal (GI) tract, all dissolution tests were conducted under non-sink conditions to promote supersaturation. The degree of deviation from sink conditions was quantified using the sink index (*SI*), a dimensionless parameter defined by the following equation:

(2) $$SI=\frac{C_{S}*V}{Dose}$$

where *C_s_* is the saturation solubility of the crystalline API (mg/mL), *V* is the volume of the dissolution medium (mL), and *Dose* is the total drug content in the tested sample (mg). In this study, a reference SI value of 0.7 was selected as a representative intermediate benchmark.

The choice of pH 4.5 acetate buffer was made to balance the solubility profiles of both APIs. While CBZ exhibits minimal pH-dependent solubility across the physiological pH range [43,44], IND shows marked pH sensitivity, with significantly lower solubility in acidic media [44]. Therefore, a dissolution medium at pH 4.5 enabled both compounds to be tested under the same intermediate non-sink conditions (SI = 0.7), thus allowing controlled induction of supersaturation and meaningful comparison of their precipitation behaviors. Additionally, it is important to note that in order to maintain constant SI and to ensure consistency across formulations, the dissolution medium volume was accordingly adjusted for each formulation based on the actual drug content of the tested tablet.

Aliquots of 2 mL were withdrawn at predetermined time intervals (5, 10, 15, 20, 30, 45, 60, 90, 120, 180, 240, 300, 360, 420, and 480 min) and immediately replaced with an equal volume of fresh pre-warmed dissolution medium to maintain constant volume and temperature. Drug concentrations in the collected samples were quantified using UV–Vis spectrophotometry at 318 nm for IND and 284 nm for CBZ. All dissolution studies were carried out in triplicate, and results are reported as mean values ± standard deviation (SD).

### 2.8. Stability Studies

Tablets from each formulation system, for both APIs, were stored in open glass vials under controlled humidity conditions (60 ± 5% RH) at room temperature in desiccators. It should be noted that, in accordance with ICH guidelines, long-term stability testing is typically conducted on the final drug product under conditions of 25 °C ± 2 °C/60% RH ± 5% RH for 12 months or under accelerated conditions of 40 °C ± 2 °C/75% RH ± 5% RH for 6 months. However, these studies are designed for the finished dosage form stored in its intended primary packaging (e.g., alu-alu blisters or high-density polyethylene bottles), which provide protection against environmental stressors such as moisture and light. In contrast, in the present study, the tablets were stored for a shorter duration (3 months) in open glass vials, without protective commercial packaging. This setup represents a worst-case scenario for moisture uptake and physical instability, thereby enhancing the sensitivity of the study to detect even subtle differences in the physical stability and supersaturation performance of the formulations [13]. Such open-dish storage conditions are widely employed during early-stage formulation development as a predictive tool for identifying robust amorphous systems prior to packaging design and optimization. After a storage period of three months, the tablets were evaluated to assess potential changes in their solid-state characteristics and drug release performance. Specifically, tensile strength, disintegration time, in vitro dissolution behavior, and physical state were examined using the same instrumentation and methodologies previously described. This stability assessment aimed not only to detect possible recrystallization or transformations in the amorphous phase of the APIs but also to evaluate the integrity of the release profile and mechanical performance of the tablets following prolonged exposure to high humidity.

### 2.9. Statistical Analysis

Statistical significance in the differences between dissolution profiles was assessed using Student’s *t*-test. A *p*-value (p*) less than 0.05 was considered indicative of a statistically significant difference. All statistical analyses were conducted using SPSS software, version 15.0 (SPSS Inc., Chicago, IL, USA). Additionally, the area under the curve (AUC_(0→t)_) for the in vitro dissolution profiles was calculated using OriginLab software version 7.5 (OriginLab Corporation, Northampton, MA, USA).

## 3. Results and Discussion

### 3.1. Thermal Stability Profile of Raw Materials

Given that the QC method was selected for the preparation of ASDs, it was essential to evaluate the thermal stability of the raw materials prior to formulation development. Fusion-based techniques, such as QC, typically involve heating the drug-polymer mixture above the melting point or Tg of the components, followed by rapid cooling to trap the API in an amorphous state. As this process exposes the materials to elevated temperatures, it may not be suitable for thermally labile compounds. Therefore, a preliminary assessment of the thermal behavior of the individual components using TGA is critical to ensure their stability under processing conditions.

**Figure 1:**
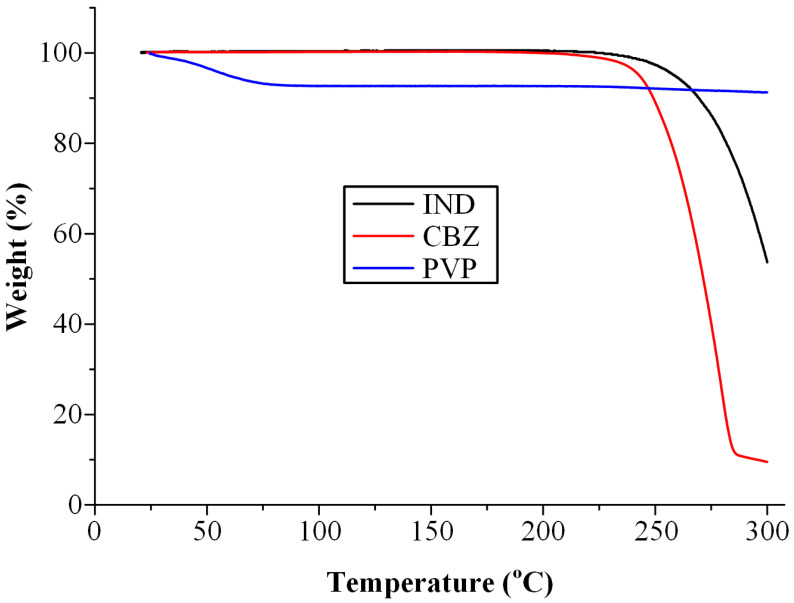
TGA thermograms of raw materials.

The TGA thermograms, shown in Figure 1, reveal that IND exhibits thermal stability up to approximately 230 °C, while CBZ remains stable up to around 220 °C. These findings are consistent with previously reported data [45,46], confirming the suitability of both APIs for processing via melt-based techniques. Regarding the polymeric carrier, PVP, a mass loss of approximately 7% *w*/*w* was observed below 100 °C, which is attributed to the evaporation of residual moisture rather than thermal degradation. No decomposition was detected within the processing temperature range. Overall, the results confirm the thermal suitability of IND, CBZ, and PVP for processing via the selected QC method, indicating that the ASD formulations can be prepared under the applied thermal conditions without compromising the thermal stability of the constituent materials. It should be noted that while TGA may overpredict degradation due to differences in heating profiles and testing atmospheres [47], in this study, it was used solely to confirm that the selected preparation temperatures (190 °C for IND and 210 °C for CBZ) were below the degradation thresholds of each API.

### 3.2. Evaluation of ASDs

#### 3.2.1. DSC Results

Figures S1a and S2a (Supplementary Material) present the DSC thermograms of the neat APIs, IND and CBZ, respectively, along with PVP and their corresponding ASDs formulated at varying drug-to-polymer weight ratios (10:90, 20:80, and 40:60 *w*/*w*). The thermogram of PVP exhibits a broad endothermic peak below 100 °C, attributed to the evaporation of absorbed moisture, which is consistent with the hydrophilic nature of the polymer. This observation aligns closely with the results obtained from TGA, which also indicated moisture loss occurring at temperatures below 100 °C.

For neat IND (Figure S1a), a single sharp endothermic peak corresponding to the melting of the γ-crystalline form was observed at 163.5 °C (onset at 159.9 °C), with an associated enthalpy of fusion (ΔH_f_) of 126 J/g. In the case of CBZ (Figure S2a), two distinct endothermic events were detected. The first, a minor peak at 175.8 °C (onset at 173.9 °C, ΔH_f_ = 14.28 J/g), corresponds to the polymorphic transformation from form III to form I. This was followed by a prominent melting peak at 193.4 °C (onset at 190.6 °C, ΔH_f_ = 132.7 J/g), indicative of the melting of form I crystals. In contrast to the neat APIs, the thermograms of all ASD formulations showed no detectable melting endotherms, regardless of the drug-to-polymer ratio. The absence of thermal transitions characteristic of the crystalline forms suggests that both IND and CBZ were successfully converted into an amorphous state within the PVP matrix across all compositions tested.

Furthermore, DSC analysis further revealed that Tg values decreased progressively with increasing drug loading for both IND- and CBZ-based ASDs. Specifically, Tg values for IND–PVP ASDs were 69.5 °C, 67.8 °C, and 64.3 °C at drug-to-polymer ratios of 10:90, 20:80, and 40:60 *w*/*w*, respectively, while CBZ–PVP ASDs exhibited Tg values of 51.6 °C, 50.1 °C, and 48.7 °C at the same ratios. This reduction in Tg is consistent with the plasticizing effect of the APIs on PVP, leading to increased molecular mobility within the amorphous matrix and potentially reduced resistance to recrystallization [48].

#### 3.2.2. Evaluation of Molecular Interactions via ATR-FTIR

In addition to the thermophysical and physicochemical characteristics of ASDs, the presence of intermolecular interactions between the API and the polymeric carrier is widely recognized as a critical factor influencing the formation, physical stability, and overall performance of the system [11,49]. Hence, following the evaluation of the thermal properties and physical state of the prepared ASDs, it is essential to gain insight into the nature of these interactions. Such understanding can provide valuable information regarding the stabilization mechanisms of the amorphous APIs within the polymer matrix. To this end, ATR-FTIR spectroscopy was employed to investigate the potential interactions between PVP and the model APIs.

Figure S1b illustrates the ATR-FTIR spectra of pure IND, the PMs, and the ASDs across all examined drug-to-polymer ratios. In the case of crystalline IND (γ-polymorph), the most prominent absorption bands are observed at 1710 cm^−1^ and 1685 cm^−1^, corresponding to the stretching vibrations of the carboxylic acid carbonyl and the amide carbonyl groups, respectively. These bands are characteristic of the intermolecular carboxylic acid homo-synthon interactions typical of the γ-form of IND. Additional notable peaks include those at 1257 cm^−1^, attributed to asymmetric aromatic –O–C stretching, and at 1086 cm^−1^, associated with symmetric aromatic –O–H stretching. The amorphous form of IND displays a distinct spectral profile. Specifically, a shoulder at 1733 cm^−1^ is evident, representing non-hydrogen-bonded carboxylic acid groups, along with a peak at 1705 cm^−1^ corresponding to the asymmetric C=O stretching of a cyclic dimer. A peak at 1678 cm^−1^ is assigned to the stretching of the benzoyl carbonyl group, while a band at 1581 cm^−1^ is associated with indole ring vibrations. These spectral features are in agreement with previously published data and serve as clear markers of the amorphous state. Regarding PVP, its ATR-FTIR spectrum exhibits characteristic absorption bands consistent with its chemical structure. Key peaks include a broad band at 3425 cm^−1^ attributed to adsorbed water, a peak at 2968 cm^−1^ corresponding to C–H stretching, and a strong band at 1648 cm^−1^ associated with C=O stretching vibrations of the lactam group. Additional signals at 1461 and 1420 cm^−1^ are assigned to CH_2_ bending (δCH_2_), and a peak at 1288 cm^−1^ is attributed to C–N stretching. In contrast, the ASD spectra demonstrated noticeable deviations from those of the corresponding PMs, suggesting the establishment of molecular interactions between functional groups of the drug and the polymer. Notably, the carbonyl stretching vibration of PVP, initially recorded at 1651 cm^−1^ in the PMs, exhibited a consistent blue shift in the ASD formulations—shifting to 1664, 1660, and 1668 cm^−1^ in the 10:90, 20:80, and 40:60 IND:PVP ratios, respectively. These shifts are indicative of hydrogen bonding interactions between the carbonyl groups of IND and those of PVP. Considering that PVP contains a single hydrogen bond acceptor site, namely the lactam carbonyl, the observed spectral changes are consistent with previously reported interaction patterns in similar amorphous systems [50]. Such interactions are known to contribute to the stabilization of the amorphous phase by reducing molecular mobility and inhibiting recrystallization.

Figure S2b presents the ATR-FTIR spectra for CBZ’s PMs and ASDs across all previously studied drug-to-polymer ratios. The spectrum of crystalline CBZ corresponds to its polymorphic form III and exhibits several well-defined characteristic peaks. Specifically, absorption bands at 3464 cm^−1^ and 3160 cm^−1^ are attributed to N–H stretching vibrations of the amide group, while the band at 1672 cm^−1^ corresponds to C=O stretching. Additionally, a doublet observed in the 1590–1605 cm^−1^ region is assigned to aromatic C=C stretching vibrations and –NH deformation. Peaks at 1308 and 1380 cm^−1^ are associated with C–N stretching of the amide group, and the absorption at 760 cm^−1^ reflects out-of-plane bending vibrations of aromatic C–H bonds. In contrast, the ATR-FTIR spectrum of amorphous CBZ reveals broadening and shifting of key functional group vibrations, consistent with the disruption of long-range molecular order. A broad absorption band centered at approximately 3480 cm^−1^, along with a wide band spanning the 3000–3400 cm^−1^ region, indicates altered N–H stretching patterns due to the breakdown of hydrogen bonding networks present in the crystalline state. Furthermore, the C–N stretching of the amide group shifts to 1244 cm^−1^. These spectral modifications confirm the amorphization of CBZ and are in agreement with previous reports [51,52]. For all PMs, the recorded ATR-FTIR spectra appeared as simple superimpositions of the spectra of the individual crystalline CBZ and PVP components, irrespective of the CBZ-PVP ratio. This observation suggests that no significant molecular interactions were established between the crystalline API and the polymer during the physical mixing stage, prior to the melt-quench process. Furthermore, the spectral features confirm that CBZ retained its crystalline structure throughout the mixing phase. In the case of CBZ–PVP ASDs, clear spectral evidence supports the presence of specific molecular interactions between the drug and the polymer. A key indicator is the absence of the characteristic N–H stretching band of amorphous CBZ at 3480 cm^−1^ in all ASD spectra. This disappearance suggests a disruption of the original hydrogen bonding environment within CBZ, likely due to the formation of new interactions with PVP. Additional confirmation is provided by the observed shift in the carbonyl stretching vibration of PVP, which appears at 1652 cm^−1^ in the PMs but shifts to lower wavenumbers in the ASD systems—specifically to 1647 cm^−1^ in the 10:90 and 20:80 CBZ-PVP formulations and to 1642 cm^−1^ in the 40:60 formulation. This shift is indicative of hydrogen bonding between the carbonyl groups of PVP and the functional moieties of CBZ. The combined spectral changes point to the formation of stabilizing intermolecular interactions in the amorphous matrix, which likely contribute to the molecular dispersion of CBZ within PVP and enhance the physical stability of the amorphous form by inhibiting recrystallization.

It should be noted that the molecular interactions identified by ATR-FTIR, such as hydrogen bonding between the APIs and PVP, are non-covalent in nature and do not alter the intrinsic structure-activity relationship (SAR) of the drug. ASDs are primarily designed to enhance bioavailability by improving solubility and dissolution, while the pharmacological activity of the API, determined by its interactions with biological targets, remains unchanged [53,54]. This distinction has been confirmed by in vivo studies, where CBZ–PVP ASDs demonstrated enhanced oral bioavailability without affecting CBZ’s pharmacological effect [55].

### 3.3. Characterization of the Prepared Tablets Immediately After Preparation

#### 3.3.1. Physical State Evaluation

Before assessing the influence of formulation and processing parameters—such as compression pressure (50–250 MPa), dwell time (5–60 s), drug-to-polymer ratio, and ASD loading—on the pharmaco-technical performance of the tablets (i.e., tensile strength and disintegration time) and their dissolution behavior, it is essential to first confirm that the amorphous state of the API was maintained following the preparation of the ASD and the tableting process. To this end, pXRD analysis was conducted on all neat ASDs and the tablet formulations immediately after compression.

The pXRD patterns of the neat APIs (IND and CBZ), the PVP, and their respective ASDs at different drug-to-polymer weight ratios (10:90, 20:80, and 40:60 *w*/*w*) are presented in Figures S1c and S2c. As illustrated in Figure S1c, PVP exhibited two broad halos within the 2θ range of 5–45°, confirming its inherently amorphous nature. In contrast, IND showed a series of sharp and well-defined peaks at 2θ values of 11.6°, 16.7°, 19.6°, 21.8°, 26.6°, and 29.3°, indicative of its crystalline form and in alignment with previously published data [56]. Likewise, the diffraction pattern of CBZ, shown in Figure S2c, revealed pronounced peaks at 2θ = 15.3°, 15.8°, 17.1°, 24.9°, 27.2°, and 27.5°, which are characteristic of its crystalline structure and consistent with literature reports [57]. In contrast, the pXRD profiles of all ASD formulations prepared with IND and CBZ displayed no observable crystalline peaks across all examined compositions. The complete absence of sharp peaks in these spectra confirms that both APIs were successfully transformed into the amorphous state, regardless of the drug loading. These results strongly prove, in conjunction with the DSC findings (presented in Section 3.2.1), that full amorphization was achieved in all ASD systems evaluated before tableting.

**Figure 2:**
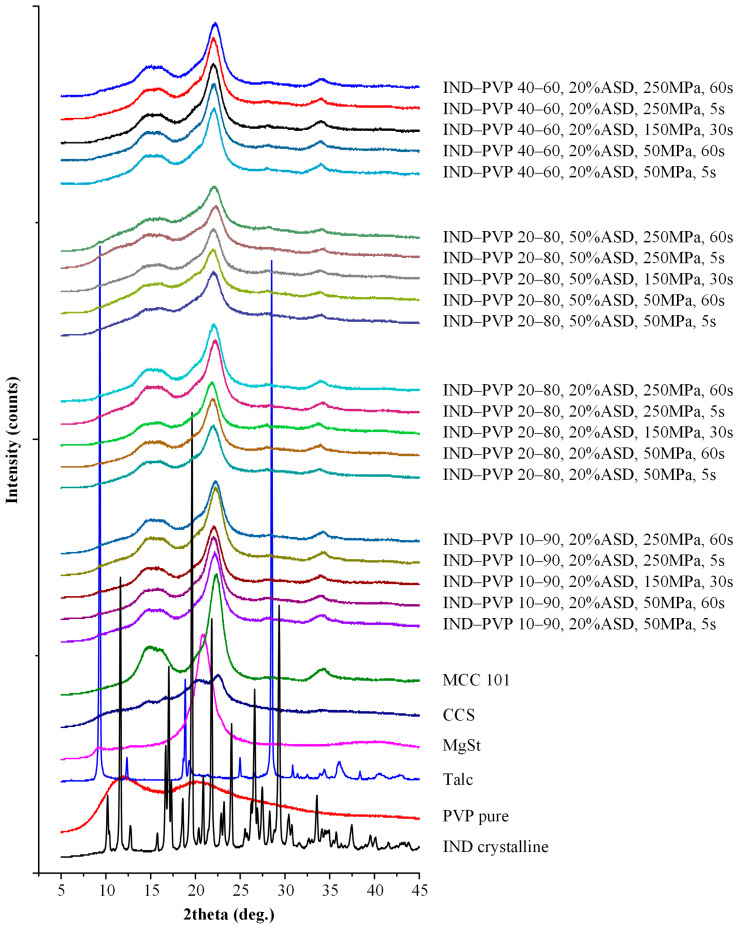
pXRD patterns of IND–PVP tablets immediately after compression, prepared at varying API-to-polymer ratios (10:90, 20:80, and 40:60 *w*/*w*) and ASD loadings (20% and 50% *w*/*w*), subjected to different compaction pressures (50, 150, and 250 MPa) and dwell times (5, 30, and 60 s). All tablets were evaluated immediately after preparation.

**Figure 3:**
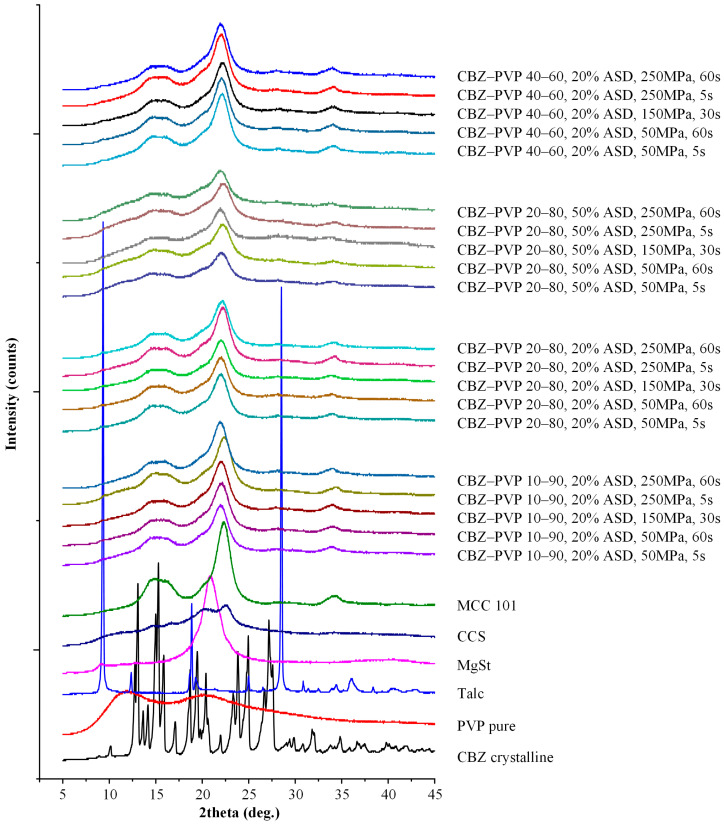
pXRD patterns of CBZ–PVP tablets immediately after compression, prepared at varying API-to-polymer ratios (10:90, 20:80, and 40:60 *w*/*w*) and ASD loadings (20% and 50% *w*/*w*), subjected to different compaction pressures (50, 150, and 250 MPa) and dwell times (5, 30, and 60 s). All tablets were evaluated immediately after preparation.

With regard to the prepared tablets, pXRD was selected in order to evaluate the presence of residual drug crystallinity. This technique was selected instead of DSC since the latter has shown to induce in situ amorphization during the heating step, a process that, in many cases, obscures the detection of residual crystallinity in the samples. Figure 2 and Figure 3 present the pXRD patterns of all tablet components (PVP, IND, CBZ, and additional tablets’ excipients), as well as the diffraction spectra of the final tablet formulations across the various API-to-polymer ratios (10:90, 20:80, 40:60 *w*/*w*) and ASD loadings (20% and 50% *w*/*w*) under all tested compaction conditions.

MCC 101, a semi-crystalline material, showed a broad amorphous halo in the 2θ range of 12.5–17.5°, a defined crystalline peak at 2θ = 22.4°, and a smaller broad peak near 2θ = 34°. MgSt displayed a minor peak at 2θ = 9.2° and a broad feature from 16–25°. Talc, a well-known crystalline compound, exhibited multiple distinct peaks, with the most prominent at 2θ = 9.4°, 18.9°, and 28.5°, in agreement with previously reported spectra [58,59,60,61].

Critically, the pXRD patterns of the ASD-based tablets for both IND and CBZ demonstrated no observable crystalline peaks attributable to the APIs across all formulations and compaction conditions. This confirms that the APIs remained in the amorphous state after tableting, irrespective of the drug-to-polymer ratio, ASD loading, or the applied compression pressure and dwell time.

#### 3.3.2. Tensile Strength Evaluation

**Figure 4:**
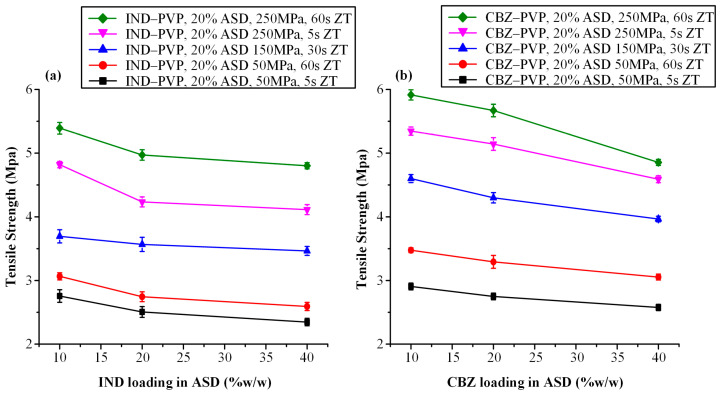
Effect of compression parameters on tablet formulations containing a fixed ASD loading of 20% *w*/*w*: (**a**) IND–PVP tablets at varying API-to-polymer ratios, and (**b**) CBZ–PVP tablets at varying API-to-polymer ratios. All tablets were evaluated at zero time (ZT), immediately after preparation.

Figure 4 presents the influence of compaction parameters on the tensile strength of tablets containing a fixed ASD loading of 20% *w*/*w*, formulated at varying API-to-polymer ratios for IND–PVP (Figure 4a) and CBZ–PVP (Figure 4b) systems. In the IND-based formulations, an increase in both compression pressure and dwell time led to higher tensile strength across all tested API-polymer ratios. Notably, compression pressure exhibited a more pronounced effect than dwell time; at constant pressure, increases in dwell time (from 5 to 60 s) resulted in modest tensile strength gains, whereas at constant dwell time, a substantial improvement in mechanical strength was observed with increasing compression pressure.

A clear inverse relationship was also observed between the API-to-polymer ratio and tensile strength: as the proportion of IND in the ASD increased from 10:90 to 40:60 *w*/*w*, tablet tensile strength progressively declined. This finding suggests a higher degree of brittleness with increasing drug loading. This observation aligns with previously reported trends, which have shown that greater drug content correlates with microcrack formation and reduced mechanical integrity [26,62,63].

Furthermore, the observed tensile strength’s decrease can be attributed to the reduction in the polymer fraction, which in turn impairs the tablet’s ability to plastically deform and form strong interparticulate bonds. These observations align with the well-established understanding that both interparticulate bonding area and bonding strength contribute to tablet tensile strength [20,64]. Bonding area is influenced by particle characteristics—such as size, morphology, and compression pressure—while bonding strength is governed by the chemical nature of the materials and the extent of intermolecular interactions [65,66]. PVP, known for its high ductility and capacity for plastic deformation, plays a crucial role in promoting tabletability. Under compression, PVP molecules undergo backbone rotation via dihedral angle transitions, facilitating segmental mobility and the creation of extensive bonding interfaces [67]. This phenomenon promotes tight junctions between adjacent particles, allowing for intermolecular hydrogen bonding and the development of strong compacts. However, as the proportion of drug in the ASD increases, the amount of plastically deforming material diminishes, leading to a reduction in bonding area and cohesion and resulting in weaker, more brittle tablets.

Similar mechanical behavior was observed in CBZ-based tablets (Figure 4b), where tensile strength increased predominantly with compaction pressure and, to a lesser extent, with dwell time. A progressive reduction in tensile strength was again noted with increasing CBZ-to-PVP ratios. This may be attributed to the inherently lower compressibility and higher brittleness of amorphous CBZ compared to PVP, as was similarly observed in the IND-based formulations.

**Figure 5:**
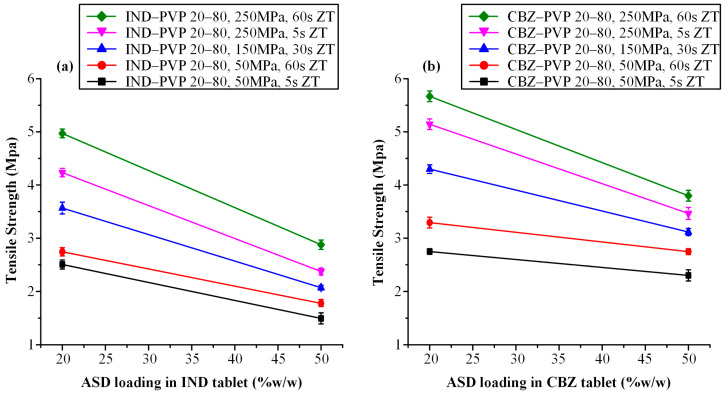
Effect of compression parameters on tablet formulations containing ASDs with a fixed API-to-polymer ratio of 20:80 *w*/*w*: (**a**) IND–PVP tablets and (**b**) CBZ–PVP tablets, each prepared with varying ASD loadings. All tablets were evaluated at zero time (ZT) after preparation.

Figure 5a,b depict the influence of ASD loading (20% and 50% *w*/*w*) on the tensile strength of tablets containing IND–PVP and CBZ–PVP, respectively, across varying compaction pressures and dwell times. For both APIs, a clear inverse relationship was observed: as the ASD content increased, tensile strength decreased. This trend is consistent with previous findings by Sauer et al. [68], who reported that formulations with high ASD loadings exhibited reduced compactibility and mechanical strength. At elevated compression pressures, the energy applied during compression was not fully accommodated by deformation of the ASD particles. These findings underscore the need for careful optimization of ASD content in conjunction with suitable binder or disintegrant selection to ensure adequate tablet robustness and mechanical performance.

It is also noteworthy that regardless of the API-to-polymer ratio or the ASD loading in each tablet, CBZ-based formulations consistently exhibited higher tensile strength compared to their IND-based counterparts when compressed under identical conditions (i.e., same compaction pressure and dwell time). This observation underscores the influence of the specific API on the mechanical behavior of ASD tablets. On a molecular level, several mechanisms may account for these differences, including variations in the extent of free volume reduction due to “hole-filling” by the smaller API molecule, differences in sub-glass transition (β-relaxation) mobility, and the nature or strength of API–polymer interactions [62]. These factors collectively influence the molecular mobility and packing efficiency within the ASD matrix, ultimately affecting the mechanical integrity of the resulting tablets.

It should be noted that beyond the above-mentioned properties, other physicochemical characteristics of the APIs, such as particle size distribution, bulk density, and flowability parameters (e.g., Hausner’s ratio), could also influence downstream processing and, in particular, the tensile strength of ASD-based tablets. However, these factors were not investigated in the present work, as they were considered to fall outside the scope of this study.

#### 3.3.3. Disintegration Time Evaluation

**Figure 6:**
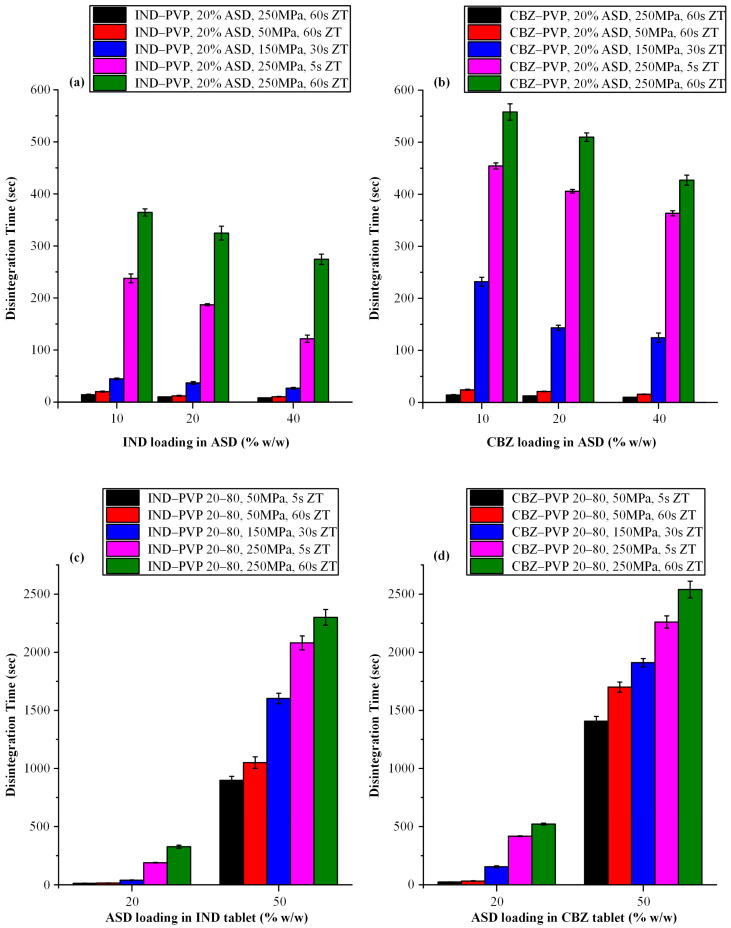
Effect of compression parameters on disintegration time of tablets containing (**a**) IND-based ASDs and (**b**) CBZ-based ASDs with a fixed ASD loading of 20% *w*/*w* and varying API-to-polymer ratios, as well as tablets with a fixed API-to-polymer ratio of 20:80 and increased ASD loading of 50% *w*/*w*: (**c**) IND-based ASDs and (**d**) CBZ-based ASDs. All tablets were evaluated at zero time (ZT), immediately after preparation.

Following the evaluation of tensile strength, the impact of compression parameters on the disintegration behavior of tablets was investigated, focusing on formulations containing varying API-to-polymer ratios and ASD loadings for both IND and CBZ systems (Figure 6).

With respect to the influence of the IND-to-PVP ratio on disintegration behavior (Figure 6a), a clear trend was observed: as the proportion of IND increased from 10:90 to 40:60 *w*/*w*, disintegration time decreased progressively. This trend is consistent with the corresponding reduction in tensile strength observed across the same formulations. The shorter disintegration times associated with higher drug content can be attributed to two primary factors. Primarily, a lower polymer content is associated with decreased tensile strength, which likely corresponds to decreased porosity. This reduction in compactness can enhance porosity, which in turn facilitates more efficient water penetration and promotes faster disintegration. Secondly, PVP—a hydrophilic polymer commonly used in ASD formulations—has been reported to exhibit a gelling effect upon hydration. This gel layer may act as a diffusion barrier, impeding the penetration of water and consequently delaying disintegration and dissolution [69]. Several studies have noted that formulations with high PVP content may exhibit prolonged disintegration times due to this gel formation [70]. Therefore, formulations with higher API content (and thus lower polymer fractions) are less affected by this phenomenon, enabling faster disintegration. In the case of CBZ-containing tablets having 20% *w*/*w* of ASD, Figure 6b demonstrates a comparable pattern to that observed in the IND containing systems, wherein an increase in the API-to-polymer ratio is associated with a progressive reduction in disintegration time. These findings are consistent with previous literature [25,71].

In general, it should be noted that beyond the gelation tendency of PVP, the hydrophilic character of the polymer plays a critical role in determining the disintegration time of ASD tablets and the manner in which variations in the polymer-to-API ratio influence this parameter. The systematic study conducted by Zhang et al. [25] investigated the effect of polymer type (two hydrophilic polymers, copovidone, PVPVA, and hydroxypropyl methyl cellulose, HPMC, and one relatively hydrophobic polymer, HPMCAS) and polymer–drug ratio on the disintegration of ASD tablets. Their findings demonstrated that increasing the polymer–drug ratio led to a corresponding increase in disintegration time for tablets based on hydrophilic polymers. In contrast, for HPMCAS-based systems, disintegration time remained short and largely unaffected, highlighting the influence of polymer hydrophilicity on tablet performance. These differences were attributed to variations in polymer hydrophilicity and the viscosity of their aqueous solutions. Therefore, given the highly hydrophilic nature of PVP, the polymer employed as the ASD carrier in this study, the observed results can be considered consistent with previous findings.

A noteworthy observation arises when comparing the disintegration behavior of IND- and CBZ-containing tablets. As illustrated in Figure 6a,b, CBZ-containing formulations consistently exhibit longer disintegration times than their IND counterparts, even when prepared with identical API-to-polymer ratios, ASD loadings, and compaction conditions. One possible explanation for this trend lies in the superior mechanical strength of the CBZ–PVP tablets. It is well established that higher tensile strength is typically associated with reduced tablet porosity, which in turn impedes water penetration and delays the onset of disintegration. Thus, the prolonged disintegration times observed in the CBZ-containing formulations may be attributed, at least in part, to their enhanced compactness [72].

Figure 6c,d, corresponding to IND- and CBZ-containing tablets, respectively, depict the influence of varying ASD loadings (20% and 50% *w*/*w*) on tablet disintegration time across different compaction pressures and dwell times. For both APIs, a substantial increase in disintegration time is observed with higher ASD loading. This behavior can be primarily attributed to the increased PVP content in the formulations with higher ASD loadings, which enhances the polymer’s gel-forming tendency upon hydration and creates a diffusion barrier, thereby delaying tablets’ disintegration time.

#### 3.3.4. Supersaturated Dissolution Studies

ASDs have emerged as a prominent strategy for enhancing the oral bioavailability of poorly water-soluble drugs, owing to the inherently higher kinetic solubility and dissolution rates of the amorphous APIs as compared to their crystalline counterparts. This enhanced performance is attributed to the elevated Gibbs free energy of the amorphous form. Specifically, given that in this case, kinetic solubility reflects the maximum apparent concentration attainable by the amorphous form under metastable conditions, in ASD formulations, this state can be sustained through liquid–liquid phase separation (LLPS) [73]. During LLPS, a colloidal drug-rich phase is generated, which acts as a reservoir that continuously replenishes the free drug fraction in solution. This reservoir effect is considered highly advantageous, as it prolongs supersaturation at levels corresponding to the amorphous solubility and thereby maximizes drug flux across the gastrointestinal membrane [74]. However, under supersaturated conditions—commonly encountered in vivo—thermodynamic instability may lead to rapid drug precipitation, which can compromise bioavailability. Therefore, the primary objective of a well-designed ASD system is twofold: (1) to ensure the physical stability of the amorphous API during storage and (2) to sustain supersaturation at the gastrointestinal absorption site upon administration.

Specifically, the in vivo behavior of ASDs reflects a complex interplay between the drug, the polymer matrix, and dynamic gastrointestinal factors, such as fluid volume, absorptive sink effects, and luminal transit conditions, which cannot be fully reproduced in vitro [75,76]. Nevertheless, dissolution studies under non-sink conditions are increasingly recognized as a meaningful tool for approximating in vivo behavior, as they allow direct evaluation of supersaturation generation, maintenance, and precipitation kinetics [32]. For this reason, non-sink dissolution testing was employed in the present work to comparatively assess the performance of the developed formulations. These tests are specifically designed to mimic in vivo environments where the solubility of the API is not maintained at sink levels, thereby enabling the investigation of supersaturation kinetics and precipitation tendencies and ultimately supporting the rational design and optimization of ASD-based drug products [77,78]. For this reason, the chosen dissolution duration (8 h) exceeded typical gastrointestinal transit times, as the primary objective of these tests was to capture the complete supersaturation–precipitation profile of the formulations. Extending the study window under *non-sink* conditions provided valuable insights into precipitation kinetics and facilitated a meaningful comparison of the relative performance of different ASD-based tablets, in line with the role of predictive dissolution testing during early-stage formulation development, as depicted by other studies as well [32].

**Figure 7:**
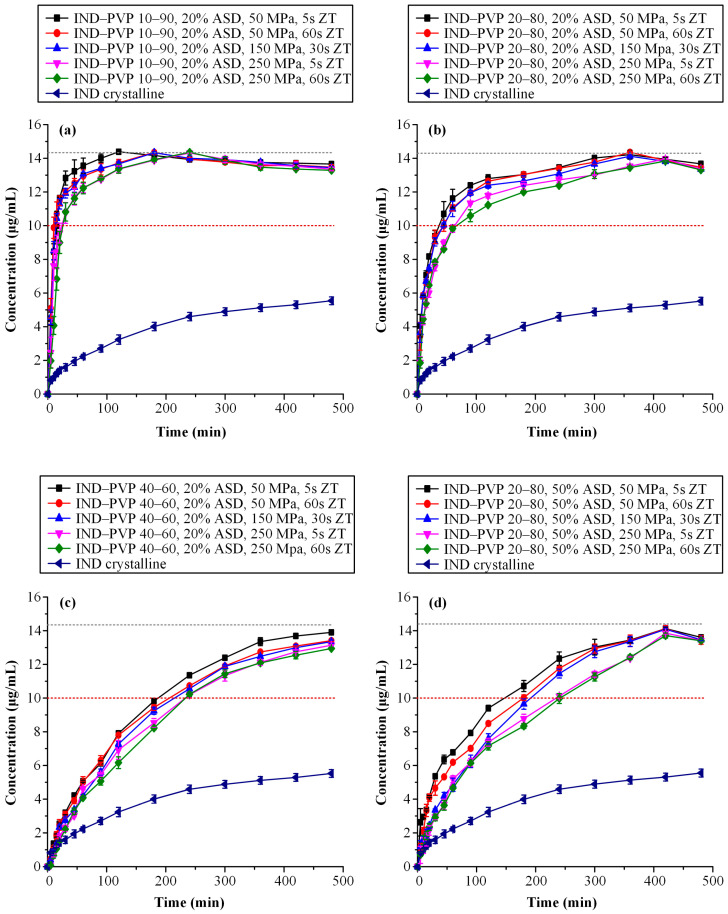
Effect of compression parameters on in vitro dissolution profiles of IND–PVP ASD tablets immediately after preparation (zero time, ZT) with 20% *w*/*w* ASD loading at API-to-polymer ratios of (**a**) 10:90, (**b**) 20:80, and (**c**) 40:60, and (**d**) with 50% *w*/*w* ASD loading at a ratio of 20:80. The gray and red dashed lines indicate the theoretical maximum concentration and crystalline IND solubility, respectively.

Figure 7 presents the dissolution profiles of IND–PVP tablets with a fixed ASD content (20% *w*/*w*), formulated at varying IND-to-PVP ratios (Figure 7a–c). The data clearly demonstrate that the drug-to-polymer ratio significantly influences the release behavior of IND. Specifically, as the IND content increases from 10% to 40% *w*/*w*, a progressive reduction in the dissolution rate is observed. This trend aligns with established dissolution patterns for ASDs, where drug loading plays a critical role in modulating release kinetics. At lower drug loadings, the polymer governs the dissolution behavior, facilitating a congruent release mechanism whereby both the drug and polymer dissolve at comparable rates [79]. In such systems, the drug release rate (${\left(\frac{dC}{dt}\right)}_{drug})$ is strongly coupled to that of the polymer (${\left(\frac{dC}{dt}\right)}_{polymer})$, which generally dissolves more rapidly. As a result, formulations with low drug loadings typically achieve rapid and complete drug release. In contrast, at higher drug loadings, this congruent release mechanism breaks down due to the formation of a drug-rich layer at the dissolution front. This layer impedes further drug diffusion, resulting in slower, diffusion-limited drug release [80]. Consequently, the reduced dissolution performance observed in the higher IND-to-PVP ratio tablets (e.g., 40:60) can be attributed to the inability of the system to maintain a congruent release profile under such conditions.

In addition to the impact of drug loading, compaction parameters—namely, compression pressure and dwell time—were also found to significantly influence supersaturation performance. Closer examination of Figure 7a–c reveals that in the case of higher drug loadings, increasing compression pressure leads to a statistically significant reduction in the dissolution rate (*p* < 0.05). In particular, for the 10:90 and 20:80 IND-to-PVP formulations, the supersaturation profiles reach similar plateau concentrations regardless of the applied compaction conditions. However, in the 40:60 system, statistically significant differences are observed, i.e., higher compression pressures result in lower maximum concentrations of dissolved IND. These findings suggest that at elevated drug loadings, the combined effects of reduced polymer content and intensified compression hinder water penetration and polymer swelling, ultimately impairing the ability of the ASD matrix to sustain the drug’s supersaturation.

To provide a more comprehensive understanding of the observed variations in supersaturation behavior, the area under the concentration–time curve (AUC_(0→t)_) was calculated for each dissolution profile and is presented in Table 2. This parameter serves as an integrative measure that captures both the extent and duration of drug supersaturation throughout the dissolution period. Given the inherent susceptibility of ASD systems to drug precipitation, AUC_(0→t)_ offers a more informative evaluation than single-point measurements or maximum concentration values, as it reflects the entire dissolution trajectory including the initial release phase, the supersaturation peak, and any subsequent decline.

The data in Table 2 confirm the previously described trends. As the IND content within the ASD matrix increases, there is a corresponding reduction in AUC_(0→t)_, indicating lower supersaturation performance at higher drug loadings. In addition, the influence of compression conditions is evident, since formulations subjected to higher compaction pressures and longer dwell times consistently show reduced AUC_(0→t)_ values. This finding highlights the negative impact of these parameters on the dissolution efficiency of ASDs.

A detailed examination of Figure 7d combined with a comparison of the AUC_(0→t)_ values between tablets containing 50% *w*/*w* ASD loading and those with 20% *w*/*w* ASD loading—both formulated at an IND-to-PVP ratio of 20:80—clearly reveals that higher ASD loading adversely impacts the overall performance of the tablets. This phenomenon may be correlated with the findings presented in the previous sections, particularly those related to disintegration behavior. It was demonstrated that increased PVP content in the formulations, associated with higher ASD loadings, enhances the polymer’s propensity to form a gel upon hydration. This gel layer acts as a diffusion barrier, impeding water penetration and consequently delaying tablet disintegration. Such delayed disintegration is likely to contribute to the reduced drug release performance observed under supersaturation conditions.

**Figure 8:**
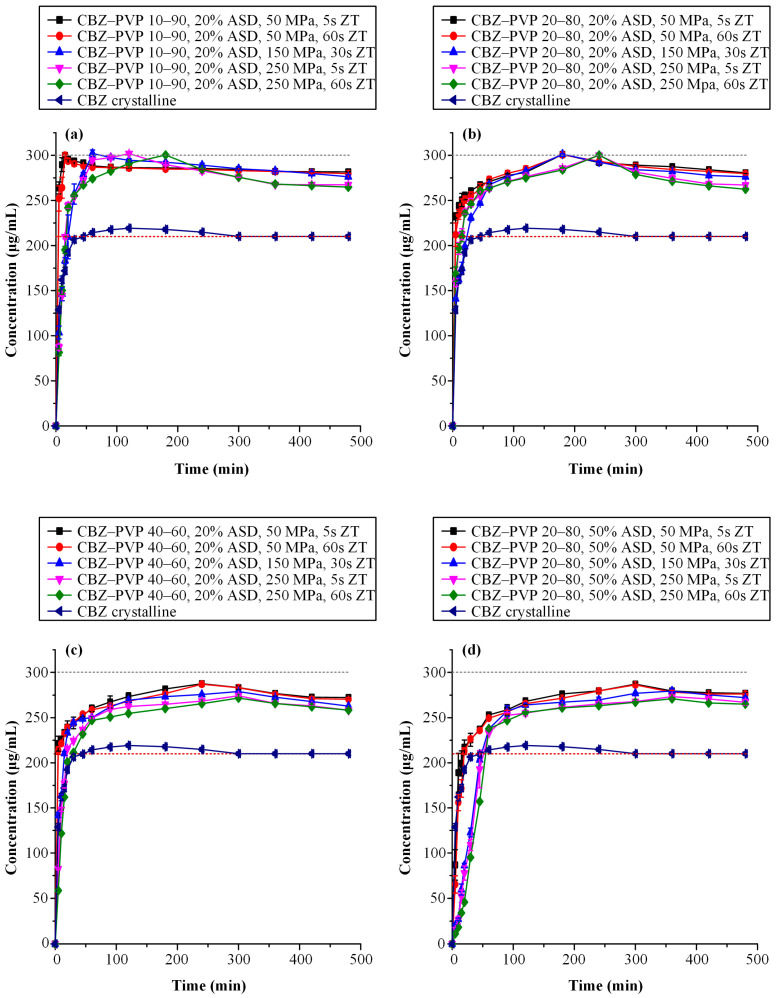
Effect of compression parameters on in vitro dissolution profiles of CBZ–PVP ASD tablets immediately after preparation (zero time, ZT) with 20% *w*/*w* ASD loading at API-to-polymer ratios of (**a**) 10:90, (**b**) 20:80, and (**c**) 40:60 and (**d**) with 50% *w*/*w* ASD loading at a ratio of 20:80. The gray and red dashed lines indicate the theoretical maximum concentration and crystalline CBZ solubility, respectively.

Figure 8 presents the dissolution study results for the CBZ-containing tablet formulations. Specifically, Figure 8a–c correspond to tablets containing a fixed ASD loading of 20% *w*/*w* and subjected to varying compaction conditions, while Figure 8d illustrates the in vitro dissolution profiles of tablets with 50% *w*/*w* ASD loading, in which all formulations maintained a constant CBZ-to-PVP ratio of 20:80.

Accordingly, Table 3 summarizes the AUC_(0→t)_ values and corresponding AUC_(0→t)_ ratios, enabling a detailed comparison of the supersaturation profiles among the evaluated CBZ-based formulations.

Based on the obtained results, a similar trend was observed for CBZ as previously described for IND containing tablet formulations. Specifically, in tablets containing 20%*w*/*w* ASD loading, an increase in drug content within the ASD formulations, as well as the application of higher compression pressures and prolonged dwell times, led to a consistent decrease in AUC_(0→t)_ values across all CBZ-to-PVP ratios. These findings confirm the negative effect of both elevated drug loading and aggressive compression parameters on the dissolution performance of CBZ-containing ASDs. Interestingly, despite CBZ being classified as a poor glass former (GFA Class I), the CBZ-containing ASD tablets demonstrated a faster and greater extent of drug release compared to their IND counterparts. This observation suggests that factors beyond glass-forming ability, such as API–polymer interactions, intrinsic molecular mobility, and drug solubility differences, may significantly influence dissolution behavior and supersaturation performance. Lastly, the negative impact of increased ASD loading on the performance of CBZ tablets—consistent with the trends observed for IND formulations—is clearly evident. Tablets containing 50% *w*/*w* ASD exhibited statistically significantly lower AUC_(0→t)_ values compared to their counterparts with 20% *w*/*w* ASD loading, highlighting the adverse effect of higher ASD content on the supersaturation performance of the final dosage forms.

### 3.4. Stability Studies Results

In the final phase of the study, all aforementioned tablet characteristics were systematically re-assessed after three months of storage under controlled conditions of ambient temperature and 60% RH.

#### 3.4.1. Physical Stability

Initially, the physical state of the tablets containing IND and CBZ after three months of storage was assessed via pXRD analysis. As shown in Figures S3 and S4 for the tablets containing the IND and CBZ ASD systems, respectively, the pXRD diffraction patterns of all tablet samples—regardless of compaction pressure, dwell time, API-to-polymer ratio, or ASD loading—remained unchanged compared to their corresponding time-zero profiles. The absence of any detectable crystalline peaks after storage confirms that both APIs remained in their amorphous form across all formulations. These findings underscore the solid-state stability of the ASD-based tablets, independent of compaction parameters, formulation composition, or storage conditions employed in this study.

Importantly, this physical stability was preserved despite the differing GFA classifications of IND (GFA III) and CBZ (GFA I), highlighting the efficacy of PVP in mitigating crystallization risk during long-term storage, even at high drug loading or high ASD content or under intense tablet compression conditions.

#### 3.4.2. Tensile Strength Evaluation After Storage

**Figure 9:**
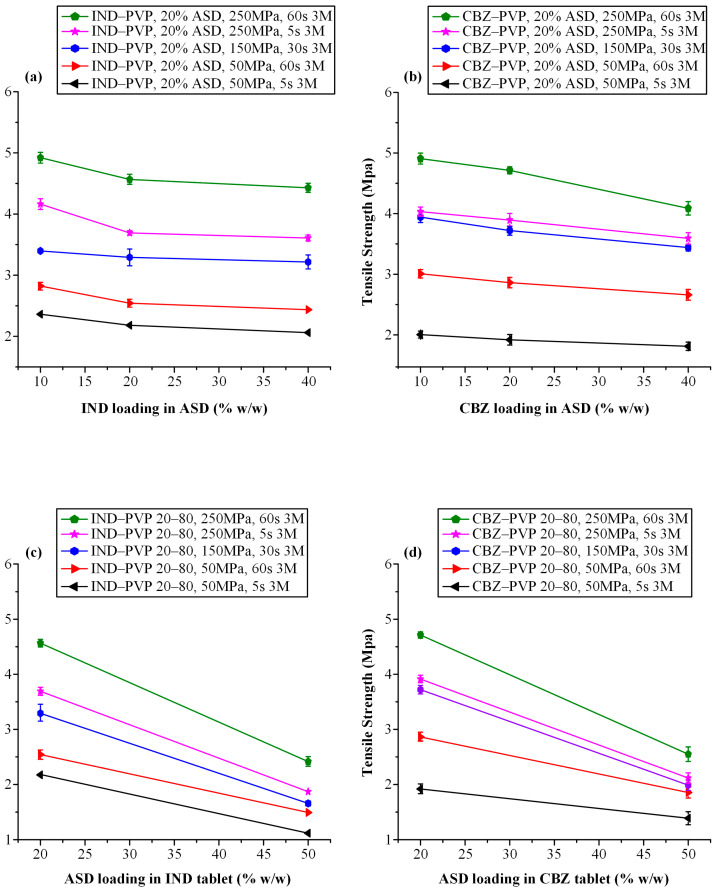
Effect of compression parameters on tensile strength of (**a**) IND and (**b**) CBZ tablets containing 20% *w*/*w* ASD loading with varying API-to-polymer ratios, as well as tablets with fixed API-to-polymer ratio (20:80) and 50% *w*/*w* ASD loading for (**c**) IND-ASDs and (**d**) CBZ-ASDs, after 3 months (3 M) of storage at 25 °C and 60% RH.

The long-term mechanical stability of ASD-based tablets was evaluated by assessing their tensile strength after three months of storage. Figure 9 presents the tensile strength values for tablets containing either IND or CBZ ASDs at varying API-to-polymer ratios, with two different ASD loadings (i.e., 20% *w*/*w* and 50% *w*/*w*). Across all tested formulations, a consistent reduction in tensile strength was observed following storage. This reduction occurred regardless of the compaction parameters applied during manufacturing, the ratio between API and polymer, or the level of ASD loading. This decline in mechanical strength suggests that storage-induced changes (likely due to moisture uptake, potential relaxation of the structure, or partial phase separation) compromise the tablet integrity over time, even in formulations that are otherwise physically stable from a solid-state perspective.

In the case of low ASD loading (20% *w*/*w*), represented in Figure 9a,b, both IND- and CBZ-containing tablets demonstrated reduced tensile strength after three months. However, the extent of this decrease was more pronounced in the CBZ formulations. This discrepancy may be attributed to differences in the inherent physicochemical properties of the two APIs, interaction with the polymer (PVP), and sensitivity to environmental stressors. CBZ, a GFA I compound, is known to be more prone to physical instability and recrystallization than IND (GFA III), and while no crystalline peaks were detected via pXRD, the greater decline in tensile strength may reflect subtle molecular-level reorganizations or weaker binding within the tablet matrix.

Interestingly, while both compression pressure and dwell time are known to influence tablet strength, the results suggest that compression pressure played a more dominant role in determining the tablets’ mechanical robustness over time. Tablets compressed at higher pressures generally maintained higher tensile strength values post-storage, indicating that stronger initial interparticulate bonding may provide some degree of resistance to storage-induced weakening. In contrast, variations in dwell time had a less pronounced impact on tensile strength retention, underscoring the idea that the magnitude of the applied pressure during tableting is a more critical determinant of long-term mechanical performance.

Figure 9c,d present the influence of storage on tablets containing a higher ASD loading (50% *w*/*w*) with a constant API-to-PVP ratio of 20:80. A significant reduction in tensile strength was observed for both IND- and CBZ-containing formulations after three months of storage, consistent with the behavior seen in tablets with lower ASD content. Notably, tablets with 50% *w*/*w* ASDs showed lower mechanical strength than those with 20% loading, underscoring the adverse effect of increased ASD content on tablet robustness. Interestingly, the decrease in mechanical strength was more pronounced in the CBZ-based tablets across all compression conditions, when compared to the corresponding zero-time values. This difference was more significant than the tensile strength reduction observed in the IND-based systems. These results highlight the fact that not only the API’s intrinsic properties but also its specific interactions with the polymer and the absorbed moisture—forming a ternary API–polymer–water network—play a key role in defining the long-term mechanical stability of ASD tablets. Such observations underscore the importance of considering molecular-level interactions and hygroscopic behavior during ASD formulation development and storage.

#### 3.4.3. Disintegration Time Evaluation After Storage

**Figure 10:**
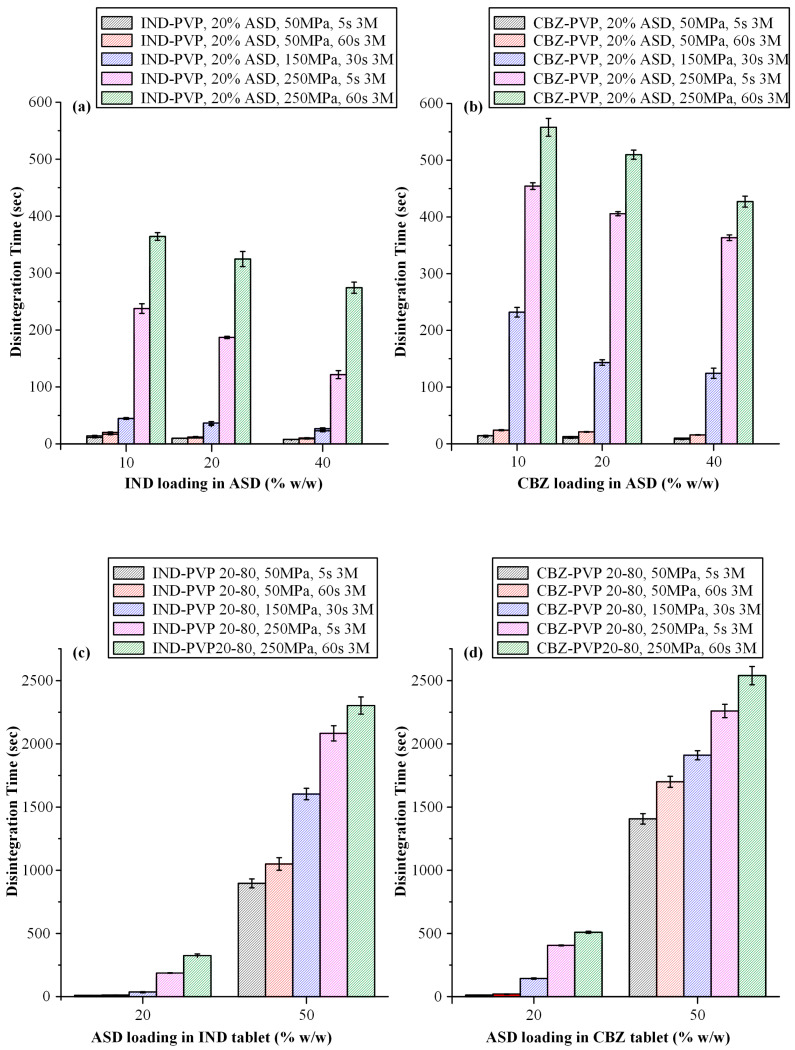
Effect of compression parameters on disintegration time of (**a**) IND and (**b**) CBZ tablets containing 20% *w*/*w* ASD loading with varying API-to-polymer ratios, as well as tablets with fixed API-to-polymer ratio (20:80) and 50% *w*/*w* ASD loading for (**c**) IND-ASDs and (**d**) CBZ-ASDs, after 3 months (3 M) of storage at 25 °C and 60% RH.

Disintegration time was further evaluated as part of the stability study. The results for tablets containing 20% *w*/*w* ASD loading with IND- and CBZ-containing systems at varying API-to-polymer ratios (10–40% *w*/*w*) are presented in Figure 10a and Figure 10b, respectively. Across all formulations, a general increase in disintegration time was observed after storage, regardless of drug loading, API, or compression conditions.

Upon closer examination of the data, it becomes evident that the increase in disintegration time was more pronounced in tablets compressed at the highest pressure (250 MPa), suggesting that intensive compression conditions significantly affect disintegration behavior over time. Furthermore, CBZ-based tablets demonstrated a more substantial increase in disintegration time relative to IND-containing formulations, highlighting the critical role of API-specific properties, variations in drug–polymer interactions, moisture sorption behavior, and structural relaxation dynamics in shaping the long-term performance of ASD-based tablets.

A comparative analysis between tablet formulations containing 50% *w*/*w* ASD loading and those with lower ASD content clearly demonstrates a more pronounced increase in disintegration time in the high-loading systems following storage. This observation is consistent with earlier findings and can be primarily attributed to the elevated concentration of the hydrophilic polymer, PVP, which enhances its gel-forming propensity upon hydration and thus acts as a diffusion barrier. Furthermore, CBZ-containing tablets appeared more susceptible to the effects of storage compared to their IND counterparts, exhibiting a statistically greater increase in disintegration time.

#### 3.4.4. Dissolution Studies After Storage

**Figure 11:**
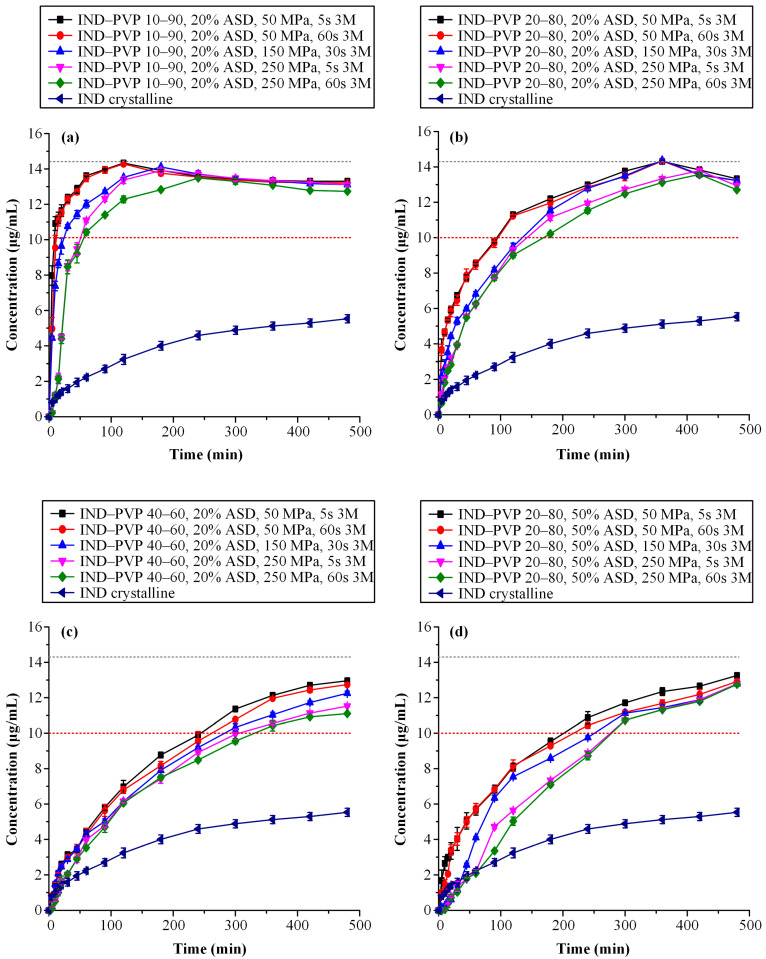
Effect of compression parameters on in vitro dissolution profiles of IND–PVP ASD tablets, after 3 months (3 M) of storage, with 20% *w*/*w* ASD loading at API-to-polymer ratios of (**a**) 10:90, (**b**) 20:80, and (**c**) 40:60 and (**d**) with 50% *w*/*w* ASD loading at a ratio of 20:80. The gray and red dashed lines indicate the theoretical maximum concentration and crystalline IND solubility, respectively.

Figure 11a–c depict the in vitro dissolution profiles of IND-containing ASD tablets having various API-to-polymer ratios, all subjected to different compaction conditions, following storage. Comparative evaluation of these profiles with those obtained immediately after preparation indicates that storage induced measurable alterations in dissolution performance. Although tablets with lower drug loadings (i.e., API-to-PVP ratios of 10:90 and 20:80) continued to reach comparable supersaturation plateau concentrations post-storage, a notable decline in the drug release rate is observed in formulations subjected to higher compaction pressures and prolonged dwell times.

Notably, formulations compressed at 250 MPa with a dwell time of 60 s exhibited the lowest drug release rate after storage. These findings suggest that intensive compaction parameters, when combined with environmental stressors such as humidity, act synergistically to impair dissolution performance. This reduction in performance could be attributed to several underlying mechanisms. Specifically, moisture uptake can induce relaxation of the polymer chains, which may weaken the intermolecular interactions between the drug and the polymer matrix. Such changes could enhance the molecular mobility of the API, increasing its susceptibility to recrystallization under supersaturated dissolution conditions.

Further confirmation of the negative effect of storage on the supersaturation behavior is provided by the AUC_(0→t)_ values presented in Table 4, which are consistently lower than those reported in Table 2 for the same formulations at time zero. This decline in AUC_(0→t)_ values is particularly pronounced in formulations with higher drug loadings and more aggressive compaction conditions, underscoring the combined influence of formulation composition and manufacturing parameters on the long-term dissolution performance of ASD-based tablets.

Finally, in the case of tablets containing 50% *w*/*w* ASD loading, the results further confirm that increased ASD content negatively impacts the dissolution performance of the formulations. A consistent pattern is again observed, since as compaction pressure and dwell time increase, the in vitro dissolution profile declines, and the ability of the formulation to maintain the API in a supersaturated state is further compromised. This finding reinforces the cumulative effect of both formulation composition and manufacturing parameters on the stability and release behavior of the amorphous drug under non-sink dissolution conditions.

**Figure 12:**
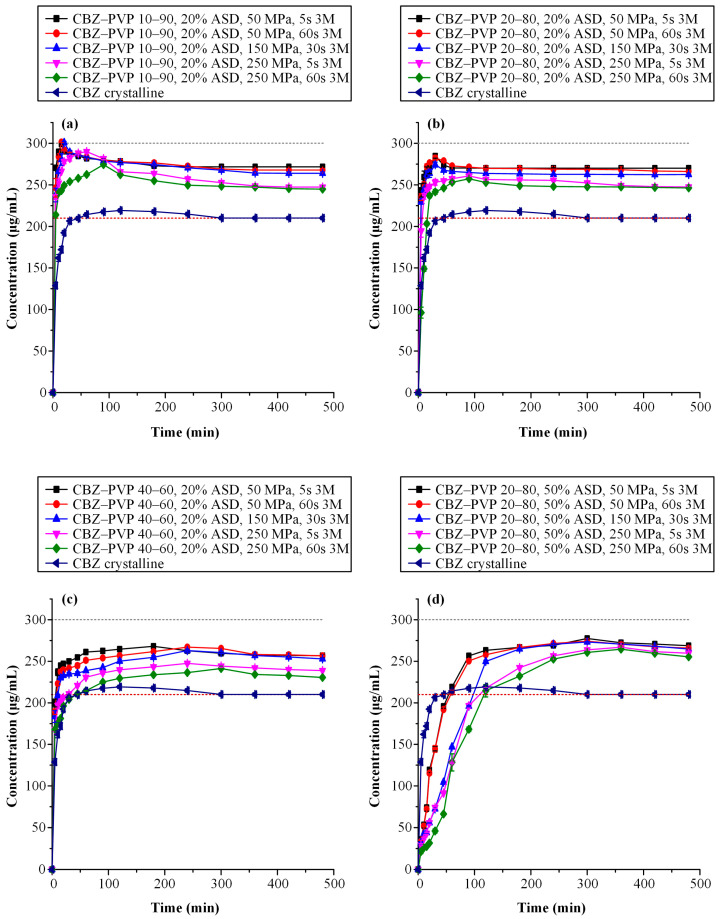
Effect of compression parameters on in vitro dissolution profiles of CBZ–PVP ASD tablets, after 3 months (3 M) of storage, with 20% *w*/*w* ASD loading at API-to-polymer ratios of (**a**) 10:90, (**b**) 20:80, and (**c**) 40:60 and (**d**) with 50% *w*/*w* ASD loading at a ratio of 20:80. The gray and red dashed lines indicate the theoretical maximum concentration and crystalline CBZ solubility, respectively.

A similar trend was observed in the dissolution profiles of CBZ-based ASD tablets, as shown in Figure 12. Specifically, formulations with increasing drug loadings (Figure 12a–c) and higher ASD content (Figure 12d) demonstrated progressively lower in vitro CBZ release. These findings confirm that the previously discussed factors—namely, drug loading and ASD loading—continue to negatively impact dissolution performance even after storage. This sustained influence is further supported by the AUC_(0→t)_ values reported in Table 5.

Comparison with the corresponding AUC_(0→t)_ values from freshly prepared formulations (Table 3) indicates that storage conditions, including environmental moisture uptake and the natural aging of the formulations, contribute to a measurable decline in overall drug release performance. It is hypothesized that these stressors may increase the molecular mobility of the API, thereby making it more susceptible to recrystallization during supersaturation-driven dissolution testing.

A close comparison of the dissolution profiles of the two APIs following storage, alongside the corresponding results obtained immediately after tablet preparation, reveals notable differences in the stability of supersaturation behavior. Specifically, CBZ-based formulations exhibited more pronounced alterations in dissolution performance across varying compression conditions and drug loadings than those containing IND. For instance, in the case of tablets containing 20% *w*/*w* ASD with a 40:60 API-to-polymer ratio, the post-storage dissolution profile showed not only a substantial reduction in the dissolution rate but also a marked decline in the maximum supersaturation level achieved, compared to both the freshly prepared counterparts and the analogous IND-containing tablets. These observations highlight the greater sensitivity of CBZ to formulation and storage-related stressors and underscore the critical influence of the API’s intrinsic properties in the performance and stability of polymeric-based ASD oral dosage forms.

The differences observed between IND- and CBZ-containing ASD tablets can be directly attributed to the distinct GFA classifications of the two APIs. Generally, it is well established that key physicochemical parameters governing glass formation include a high Tg, relative to the Tm, high equilibrium melt viscosity in combination with strong liquid behavior, and a complex molecular structure that reduces molecular mobility [81]. Based on these data, the differentiation in GFA classification is evident, with IND regarded as a good glass former (GFA Class III) and CBZ as a poor glass former (GFA Class I), a distinction that is strongly supported by their physicochemical properties [33]. Specifically, IND exhibits a comparatively higher Tg (≈42–45 °C) relative to its melting point (Tm ~160 °C), which reduces molecular mobility under ambient conditions and improves resistance against recrystallization. By contrast, CBZ presents a low Tg (≈20–25 °C) and a higher Tm (~190–193 °C), indicative of higher molecular mobility even at room temperature and a more significant tendency to recrystallization. Therefore, CBZ is expected to display more pronounced β-relaxation, corresponding to enhanced localized molecular motions, which could facilitate nucleation and accelerate recrystallization. In contrast, the higher Tg of IND would be anticipated to suppress such β-relaxation, thereby limiting local mobility and favoring improved stability.

These differences in the physicochemical properties of the APIs, which in turn determine their GFA classification, are directly linked to the behavior observed in the supersaturated dissolution studies. This observation is consistent with literature reports demonstrating a strong correlation between GFA and the supersaturation performance of amorphous drugs [82]. Specifically, it has been demonstrated that good glass formers typically achieve improved supersaturated dissolution profiles, whereas poor glass formers are limited to lower supersaturation levels. In line with these findings, the superior dissolution behavior of IND-based formulations in this study can be rationalized by its higher GFA classification.

## 4. Conclusions

This study underscores the complex interplay between formulation composition and compression parameters in determining the performance of polymer-based ASD tablets. Key formulation variables, such as the drug-to-polymer ratio and ASD loading, along with compression pressure and dwell time, were shown to significantly affect tablet tensile strength, disintegration time, and in vitro supersaturated dissolution behavior. Lower drug loadings and higher polymer content enhanced mechanical properties and supported faster disintegration and in vitro supersaturation more effectively. In contrast, increasing ASD content and applying more aggressive compression conditions were associated with diminished mechanical strength and reduced dissolution efficiency, effects that were further escalated after storage.

A direct comparison between IND (a good glass former) and CBZ (a poor glass former) further emphasized the role of API properties in shaping polymeric-based ASD tablets’ behavior. CBZ-containing formulations exhibited higher initial tensile strength and faster dissolution rates, yet their performance declined more substantially upon storage, particularly at higher drug loadings and compression forces. IND-containing systems were generally more stable under the same conditions, though they demonstrated lower initial dissolution rates. These findings highlight the necessity of tailoring formulation and processing strategies to the specific characteristics of the API in order to ensure both immediate performance and long-term stability of such polymeric-based ASD tablets.

## Figures and Tables

**Table 1:** Summary table showing the compositions of the prepared drug tablets.

	Formulation Type I	Formulation Type II
Component	% *w*/*w*	% *w*/*w*
ASD *	20	50
MCC 101	74	44
SCC	4	4
MgSt	1	1
Talc	1	1

* For both IND and CBZ, the API-to-PVP ratios in Formulation Type I were 10:90, 20:80, and 40:60 (*w*/*w*), while in Formulation Type II, the ratio was fixed at 20:80 (*w*/*w*).

**Table 2:** AUC_(0→t)_ values derived from in vitro dissolution studies of IND–PVP tablets immediately after preparation (zero time, ZT), formulated at various API-to-polymer weight ratios (10:90, 20:80, and 40:60% *w*/*w*) and ASD loadings (20% and 50% *w*/*w*). The table also includes the corresponding mean AUC_(0→t)_ ratios (i.e., AUC_(0→t)_ [sample]/AUC_(0→t)_ [IND crystalline]).

Sample ID	AUC_(0→t)_ (Mean ± SD) [μg/(mL·min) × 10^2^]	AUC_(0→t)_ Ratio (Mean)
IND–PVP 10–90, 20% ASD, 50 MPa, 5 s	65.17 ± 0.31	3.34
IND–PVP 10–90, 20% ASD, 50 MPa, 60 s	64.37 ± 0.20	3.30
IND–PVP 10–90, 20% ASD, 150 MPa, 30 s	64.04 ± 0.20	3.28
IND–PVP 10–90, 20% ASD, 250 MPa, 5 s	62.92 ± 0.21	3.22
IND–PVP 10–90, 20% ASD, 250 MPa, 60 s	62.39 ± 0.24	3.20
IND–PVP 20–80, 20% ASD, 50 MPa, 5 s	61.77 ± 0.29	3.17
IND–PVP 20–80, 20% ASD, 50 MPa, 60 s	60.93 ± 0.08	3.12
IND–PVP 20–80, 20% ASD, 150 MPa, 30 s	60.07 ± 0.18	3.08
IND–PVP 20–80, 20% ASD, 250 MPa, 5 s	57.60 ± 0.30	2.95
IND–PVP 20–80, 20% ASD, 250 MPa, 60 s	56.61 ± 0.20	2.90
IND–PVP 40–60, 20% ASD, 50 MPa, 5 s	48.46 ± 0.22	2.48
IND–PVP 40–60, 20% ASD, 50 MPa, 60 s	46.61 ± 0.38	2.39
IND–PVP 40–60, 20% ASD, 150 MPa, 30 s	45.35 ± 0.26	2.33
IND–PVP 40–60, 20% ASD, 250 MPa, 5 s	43.65 ± 0.22	2.24
IND–PVP 40–60, 20% ASD, 250 MPa, 60 s	42.78 ± 0.20	2.19
IND–PVP 20–80, 50% ASD, 50 MPa, 5 s	52.69 ± 0.32	2.70
IND–PVP 20–80, 50% ASD, 50 MPa, 60 s	50.72 ± 0.31	2.60
IND–PVP 20–80, 50% ASD, 150 MPa, 30 s	48.66 ± 0.27	2.49
IND–PVP 20–80, 50% ASD, 250 MPa, 5 s	45.54 ± 0.24	2.33
IND–PVP 20–80, 50% ASD, 250 MPa, 60 s	44.83 ± 0.20	2.30
IND crystalline	19.51 ± 0.05	1.00

**Table 3:** AUC_(0→t)_ values derived from in vitro dissolution studies of CBZ–PVP tablets immediately after preparation (zero time, ZT), formulated at various API-to-polymer weight ratios (10:90, 20:80, and 40:60% *w*/*w*) and ASD loadings (20% and 50% *w*/*w*). The table also includes the corresponding mean AUC_(0→t)_ ratios (i.e., AUC_(0→t)_ [sample]/AUC_(0→t)_ [CBZ crystalline]).

Sample ID	AUC_(0→t)_ (Mean ± SD) [μg/(mL·min) × 10^2^]	AUC_(0→t)_ Ratio (Mean)
CBZ–PVP 10–90, 20% ASD, 50 MPa, 5 s	1361 ± 1.72	1.35
CBZ–PVP 10–90, 20% ASD, 50 MPa, 60 s	1355 ± 2.09	1.34
CBZ–PVP 10–90, 20% ASD, 150 MPa, 30 s	1342 ± 1.74	1.33
CBZ–PVP 10–90, 20% ASD, 250 MPa, 5 s	1315 ± 0.95	1.31
CBZ–PVP 10–90, 20% ASD, 250 MPa, 60 s	1305 ± 3.04	1.30
CBZ–PVP 20–80, 20% ASD, 50 MPa, 5 s	1355 ± 1.25	1.34
CBZ–PVP 20–80, 20% ASD, 50 MPa, 60 s	1351 ± 1.03	1.34
CBZ–PVP 20–80, 20% ASD, 150 MPa, 30 s	1321 ± 1.91	1.32
CBZ–PVP 20–80, 20% ASD, 250 MPa, 5 s	1306 ± 2.46	1.30
CBZ–PVP 20–80, 20% ASD, 250 MPa, 60 s	1300 ± 1.50	1.29
CBZ–PVP 40–60, 20% ASD, 50 MPa, 5 s	1305 ± 3.10	1.30
CBZ–PVP 40–60, 20% ASD, 50 MPa, 60 s	1296 ± 0.71	1.29
CBZ–PVP 40–60, 20% ASD, 150 MPa, 30 s	1267 ± 1.47	1.26
CBZ–PVP 40–60, 20% ASD, 250 MPa, 5 s	1231 ± 1.23	1.22
CBZ–PVP 40–60, 20% ASD, 250 MPa, 60 s	1211 ± 4.26	1.20
CBZ–PVP 20–80, 50% ASD, 50 MPa, 5 s	1282 ± 0.80	1.27
CBZ–PVP 20–80, 50% ASD, 50 MPa, 60 s	1270 ± 1.55	1.26
CBZ–PVP 20–80, 50% ASD, 150 MPa, 30 s	1209 ± 2.48	1.20
CBZ–PVP 20–80, 50% ASD, 250 MPa, 5 s	1179 ± 4.23	1.17
CBZ–PVP 20–80, 50% ASD, 250 MPa, 60 s	1161 ± 1.08	1.15
CBZ crystalline	1008 ± 1.0	1.00

**Table 4:** AUC_(0→t)_ values derived from in vitro dissolution studies of IND–PVP tablets after 3 months (3 M) of stability, formulated at various API-to-polymer weight ratios (10:90, 20:80, and 40:60% *w*/*w*) and ASD loadings (20% and 50% *w*/*w*). The table also includes the corresponding mean AUC_0_→t ratios (i.e., AUC_(0→t)_ [sample]/AUC_(0→t)_ [IND crystalline]).

Sample ID	AUC_(0→t)_ (Mean ± SD) [μg/(mL·min) × 10^2^] 3 Μ	AUC_(0→t)_ Ratio (Mean)
IND–PVP 10–90, 20% ASD, 50 MPa, 5 s	64.05 ± 0.21	3.28
IND–PVP 10–90, 20% ASD, 50 MPa, 60 s	63.44 ± 0.23	3.25
IND–PVP 10–90, 20% ASD, 150 MPa, 30 s	62.00 ± 0.23	3.18
IND–PVP 10–90, 20% ASD, 250 MPa, 5 s	59.72 ± 0.19	3.07
IND–PVP 10–90, 20% ASD, 250 MPa, 60 s	57.26 ± 0.29	2.93
IND–PVP 20–80, 20% ASD, 50 MPa, 5 s	57.30 ± 0.22	2.94
IND–PVP 20–80, 20% ASD, 50 MPa, 60 s	56.63 ± 0.39	2.90
IND–PVP 20–80, 20% ASD, 150 MPa, 30 s	54.00 ± 0.18	2.77
IND–PVP 20–80, 20% ASD, 250 MPa, 5 s	51.09 ± 0.15	2.62
IND–PVP 20–80, 20% ASD, 250 MPa, 60 s	49.92 ± 0.10	2.56
IND–PVP 40–60, 20% ASD, 50 MPa, 5 s	43.99 ± 0.31	2.26
IND–PVP 40–60, 20% ASD, 50 MPa, 60 s	42.55 ± 0.15	2.18
IND–PVP 40–60, 20% ASD, 150 MPa, 30 s	40.31 ± 0.09	2.07
IND–PVP 40–60, 20% ASD, 250 MPa, 5 s	38.21 ± 0.17	1.96
IND–PVP 40–60, 20% ASD, 250 MPa, 60 s	37.28 ± 0.15	1.91
IND–PVP 20–80, 50% ASD, 50 MPa, 5 s	47.06 ± 0.16	2.41
IND–PVP 20–80, 50% ASD, 50 MPa, 60 s	45.36 ± 0.21	2.33
IND–PVP 20–80, 50% ASD, 150 MPa, 30 s	42.36 ± 0.02	2.17
IND–PVP 20–80, 50% ASD, 250 MPa, 5 s	38.89 ± 0.09	1.99
IND–PVP 20–80, 50% ASD, 250 MPa, 60 s	38.01 ± 0.20	1.95
IND crystalline	19.51 ± 0.05	1.00

**Table 5:** AUC_(0→t)_ values derived from in vitro dissolution studies of CBZ–PVP tablets after 3 months (3 M) of stability, formulated at various API-to-polymer weight ratios (10:90, 20:80, and 40:60% *w*/*w*) and ASD loadings (20% and 50% *w*/*w*). The table also includes the corresponding mean AUC_(0→t)_ ratios (i.e., AUC_(0→t)_ [sample]/AUC_(0→t)_ [CBZ crystalline]).

Sample ID	AUC_(0→t)_ (Mean ± SD) [μg/(mL·min) × 10^2^] 3 M	AUC_(0→t)_ Ratio (Mean)
CBZ–PVP 10–90, 20% ASD, 50 MPa, 5 s	1314 ± 3.63	1.30
CBZ–PVP 10–90, 20% ASD, 50 MPa, 60 s	1308 ± 1.93	1.30
CBZ–PVP 10–90, 20% ASD, 150 MPa, 30 s	1297 ± 2.97	1.29
CBZ–PVP 10–90, 20% ASD, 250 MPa, 5 s	1242 ± 5.68	1.23
CBZ–PVP 10–90, 20% ASD, 250 MPa, 60 s	1202 ± 3.30	1.19
CBZ–PVP 20–80, 20% ASD, 50 MPa, 5 s	1290 ± 3.12	1.28
CBZ–PVP 20–80, 20% ASD, 50 MPa, 60 s	1285 ± 2.52	1.28
CBZ–PVP 20–80, 20% ASD, 150 MPa, 30 s	1256 ± 4.94	1.25
CBZ–PVP 20–80, 20% ASD, 250 MPa, 5 s	1206 ± 2.00	1.20
CBZ–PVP 20–80, 20% ASD, 250 MPa, 60 s	1173 ± 2.74	1.16
CBZ–PVP 40–60, 20% ASD, 50 MPa, 5 s	1239 ± 2.91	1.22
CBZ–PVP 40–60, 20% ASD, 50 MPa, 60 s	1229 ± 4.76	1.22
CBZ–PVP 40–60, 20% ASD, 150 MPa, 30 s	1202 ± 0.88	1.19
CBZ–PVP 40–60, 20% ASD, 250 MPa, 5 s	1138 ± 1.52	1.13
CBZ–PVP 40–60, 20% ASD, 250 MPa, 60 s	1095 ± 3.09	1.09
CBZ–PVP 20–80, 50% ASD, 50 MPa, 5 s	1204 ± 2.00	1.19
CBZ–PVP 20–80, 50% ASD, 50 MPa, 60 s	1193 ± 3.25	1.18
CBZ–PVP 20–80, 50% ASD, 150 MPa, 30 s	1128 ± 3.03	1.12
CBZ–PVP 20–80, 50% ASD, 250 MPa, 5 s	1071 ± 3.65	1.06
CBZ–PVP 20–80, 50% ASD, 250 MPa, 60 s	1038 ± 1.70	1.03
CBZ crystalline	1008 ± 1.0	1.00

## Data Availability

The original contributions presented in this study are included in the article/Supplementary Materials. Further inquiries can be directed to the corresponding author.

## Supplementary Materials

The following supporting information can be downloaded at https://www.mdpi.com/article/10.3390/polym17182484/s1: Table S1: % Reduction in Tensile strength between zero time (ZT) and after 3 months of storage at 25 °C, 60 % RH (3M); Table S2: % Reduction in Disintegration time between zero time (ZT) and after 3 months of storage at 25 °C, 60 % RH (3M); Table S3: AUC_(0→t)_ estimated by the in vitro dissolution studies for IND-PVP tablets after 3 months of storage at 25 °C, 60% RH (3M) at API:Polymer ratios (10:90, 20:80, 40:60 %*w*/*w*) and ASD ratios (20 and 50 %*w*/*w*), along with the estimated mean AUC_(0→t)_ ratio (i.e., AUC_(0→t)_ [tablet]/AUC_(0→t)_ [IND crystalline); Table S4: AUC_(0→t)_ estimated by the in vitro dissolution studies for CBZ-PVP tablets after 3 months of storage at 25 °C, 60% RH (3M) at API:Polymer ratios (10:90, 20:80, 40:60 %*w*/*w*) and ASD ratios (20 and 50 %*w*/*w*), along with the estimated mean AUC_(0→t)_ ratio (i.e., AUC_(0→t)_ [tablet]/AUC_(0→t)_ [CBZ crystalline]; Figure S1: (a) DSC thermograms, (b) pXRD diffractograms, and (c) ATR-FTIR spectra of IND ASDs and pure materials; Figure S2: (a) DSC thermograms, (b) pXRD diffractograms, and (c) ATR-FTIR spectra of CBZ ASDs and pure materials; Figure S3: pXRD diffractograms of IND-PVP tablets after 3 months (3 M) of storage (25 °C, 60% RH); Figure S4: pXRD diffractograms of IND-PVP tablets after 3 months (3 M) of storage (25 °C, 60% RH).
