# Supplementary material for: Influence of Drug Properties, Formulation Composition, and Processing Parameters on the Stability and Dissolution Performance of Amorphous Solid Dispersions-Based Tablets
Source: Polymers (Basel). 2025 Sep 14;17(18):2484. doi: 10.3390/polym17182484 (PMC12473797; doi:10.3390/polym17182484)

Supplementary Information

Influence of Drug Properties, Formulation Composition, and Processing Parameters on the Stability and Dissolution Performance of Amorphous Solid Dispersions-Based Tablets

Ioannis Pantazos [1], Maria Poimenidou [1], Dimitrios Kouskouridas [1], Evangelos Tzaferas [1], Vasiliki Karava [1], Christos Cholevas [1], Afroditi Kapourani [1,*] and Panagiotis Barmpalexis [1,2]

[1] Laboratory of Pharmaceutical Technology, Division of Pharmaceutical Technology, School of Pharmacy, Faculty of Health Sciences, Aristotle University of Thessaloniki, 54124 Thessaloniki, Greece

[2] Natural Products Research Centre of Excellence-AUTH (NatPro-AUTH), Center for Interdisciplinary Research and Innovation (CIRI-AUTH), 57001 Thessaloniki, Greece

* Correspondence: akapourag@pharm.auth.gr

<u>Table S1</u>: % Reduction in Tensile strength between zero time (ZT) and after 3 months of storage at 25^0C, 60 % RH (3M).

**Compaction Properties**	**50MPa 5s**	**50 MPa 60s**	**150 MPa 30s**	**250MPa 5s**	**250MPa 60s**
**System**	**% Reduction in Tensile Strength from ZT to 3M (Mean ± SD)**				
IND-PVP 10-90 (20%ASD)	14.32 ± 3.17	7.93 ± 2.39	8.04 ± 2.68	13.63 ± 1.92	8.73 ± 2.23
IND-PVP 20-80 (20%ASD)	12.95 ± 3.97	7.35 ± 2.52	7.70 ± 4.28	12.83 ± 1.82	8.14 ± 2.05
IND-PVP 40-60 (20%ASD)	12.57 ± 2.24	6.04 ± 2.10	7.17 ± 3.80	12.27 ± 2.05	6.63 ± 1.79
IND-PVP 20-80 (50%ASD)	25.19 ± 5.40	15.94 ± 2.47	19.98 ± 2.80	21.27 ± 2.12	16.11 ± 3.10
CBZ-PVP 10-90 (20%ASD)	31.03 ± 2.45	13.47 ± 2.19	14.39 ± 2.26	24.56 ± 1.68	17.02 ± 1.89
CBZ-PVP 20-80 (20%ASD)	30.23 ± 1.68	13.10 ± 3.17	13.44 ± 2.42	24.29 ± 1.69	16.85 ± 1.68
CBZ-PVP 40-60 (20%ASD)	29.69 ± 2.87	12.91 ± 2.18	13.26 ± 1.15	21.76 ± 2.25	15.80 ± 2.44
CBZ-PVP 20-80 (50%ASD)	39.63 ± 5.79	32.50± 3.84	36.23 ± 3.43	38.93 ± 3.34	32.90 ± 4.02

Table S2: % Reduction in Disintegration time between zero time (ZT) and after 3 months of storage at 25^{0}C, 60 % RH (3M).

Compaction Properties	50MPa 5s	50 MPa 60s	150 MPa 30s	250MPa 5s	250MPa 60s
**System**	**% Reduction in Disintegration from time ZT to 3M**				
IND-PVP 10-90 (20%ASD)	19.05 ± 7.10	15.00 ± 6.56	15.67 ± 7.42	18.09 ± 4.77	14.45 ± 2.87
IND-PVP 20-80 (20%ASD)	18.00 ± 5.00	13.90 ± 7.18	14.55 ± 6.66	16.93 ± 2.83	13.24 ± 5.26
IND-PVP 40-60 (20%ASD)	16.25 ± 4.51	12.90 ± 6.86	13.75 ± 8.16	15.61 ± 7.33	12.03± 6.07
IND-PVP 20-80 (50%ASD)	26.39 ± 4.95	22.38 ± 4.08	23.08 ± 4.38	25.60 ± 6.70	20.84 ± 5.2
CBZ-PVP 10-90 (20%ASD)	20.93 ± 5.14	18.06 ± 4.18	18.53 ± 5.44	20.54 ± 2.54	17.56 ± 2.67
CBZ-PVP 20-80 (20%ASD)	20.26± 4.57	17.46 ± 3.62	17.67 ± 4.81	19.97 ± 1.35	16.94 ± 2.00
CBZ-PVP 40-60 (20%ASD)	20.00 ± 5.00	17.02 ± 3.06	17.43 ± 6.11	19,63 ± 2.85	16.39 ± 2.81
CBZ-PVP 20-80 (50%ASD)	31.28 ± 2.98	27.84 ± 3.34	28.97 ± 3.00	30.24 ± 3.70	25.98 ± 3.44

Table S3: AUC$_{(0\rightarrow t)}$ estimated by the *in vitro* dissolution studies for IND-PVP tablets after 3 months of storage at 25ºC, 60% RH (3M) at API:Polymer ratios (10:90 , 20:80 , 40:60 %w/w) and ASD ratios (20 and 50 %w/w), along with the estimated mean AUC$_{(0\rightarrow t)}$ ratio (i.e., AUC$_{(0\rightarrow t)}$ [tablet]/AUC$_{(0\rightarrow t)}$ [IND crystalline).

**Sample ID**	**AUC $_{(0 -> t)}$ (mean ± SD) [μg/(mL*min) x10^2] 3M**	**AUC$_{(0\rightarrow t)}$ ratio (mean)**
IND-PVP 10-90 (20%ASD) 50MPa 5s	64.05 ± 0.21	3.28
IND-PVP 10-90 (20%ASD) 50MPa 60s	63.44 ± 0.23	3.25
IND-PVP 10-90 (20%ASD) 150MPa 30s	62.00 ± 0.23	3.18
IND-PVP 10-90 (20%ASD) 250MPa 5s	59.72 ± 0.19	3.07
IND-PVP 10-90 (20% ASD) 250MPa 60s	57.26 ± 0.29	2.93
IND-PVP 20-80 (20%ASD) 50MPa 5s	57.30 ± 0.22	2.94
IND-PVP 20-80 (20%ASD) 50MPa 60s	56.63 ± 0.39	2.90
IND-PVP 20-80 (20%ASD) 150MPa 30s	54.00 ± 0.18	2.77
IND-PVP 20-80 (20%ASD) 250MPa 5s	51.09 ± 0.15	2.62
IND-PVP 20-80 (20% ASD) 250MPa 60s	49.92 ± 0.10	2.56
IND-PVP 40-60 (20%ASD) 50MPa 5s	43.99 ± 0.31	2.26
IND-PVP 40-60 (20%ASD) 50MPa 60s	42.55 ± 0.15	2.18
IND-PVP 40-60 (20%ASD) 150MPa 30s	40.31 ± 0.09	2.07
IND-PVP 40-60 (20%ASD) 250MPa 5s	38.21 ± 0.17	1.96
IND-PVP 40-60 (20% ASD) 250MPa 60s	37.28 ± 0.15	1.91
IND-PVP 20-80 (50%ASD) 50MPa 5s	47.06 ± 0.16	2.41
IND-PVP 20-80 (50%ASD) 50MPa 60s	45.36 ± 0.21	2.33
IND-PVP 20-80 (50%ASD) 150MPa 30s	42.36 ± 0.02	2.17
IND-PVP 20-80 (50%ASD) 250MPa 5s	38.89 ± 0.09	1.99
IND-PVP 20-80 (50% ASD) 250MPa 60s	38.01 ± 0.20	1.95
IND crystalline	19.51 ± 0.05	1.00

Table S4: $AUC_{(0\rightarrow t)}$ estimated by the *in vitro* dissolution studies for CBZ-PVP tablets after 3 months of storage at 25°C, 60% RH (3M) at API:Polymer ratios (10:90 , 20:80 , 40:60 %w/w) and ASD ratios (20 and 50 %w/w), along with the estimated mean $AUC_{(0\rightarrow t)}$ ratio (i.e., $AUC_{(0\rightarrow t)}$ [tablet]/$AUC_{(0\rightarrow t)}$ [CBZ crystalline).

Sample ID	$AUC_{(0 -> t)}$ (mean ± SD) [μg/(mL*min) x10^2] 3M	$AUC_{(0\rightarrow t)}$ ratio (mean)
CBZ-PVP 10-90 (20%ASD) 50MPa 5s	1314 ± 3.63	1.30
CBZ-PVP 10-90 (20%ASD) 50MPa 60s	1308 ± 1.93	1.30
CBZ-PVP 10-90 (20%ASD) 150MPa 30s	1297 ± 2.97	1.29
CBZ-PVP 10-90 (20%ASD) 250MPa 5s	1242 ± 5.68	1.23
CBZ-PVP 10-90 (20% ASD) 250MPa 60s	1202 ± 3.30	1.19
CBZ-PVP 20-80 (20%ASD) 50MPa 5s	1290 ± 3.12	1.28
CBZ-PVP 20-80 (20%ASD) 50MPa 60s	1285 ± 2.52	1.28
CBZ-PVP 20-80 (20%ASD) 150MPa 30s	1256 ± 4.94	1.25
CBZ-PVP 20-80 (20%ASD) 250MPa 5s	1206 ± 2.00	1.20
CBZ-PVP 20-80 (20% ASD) 250MPa 60s	1173 ± 2.74	1.16
CBZ-PVP 40-60 (20%ASD) 50MPa 5s	1239 ± 2.91	1.22
CBZ-PVP 40-60 (20%ASD) 50MPa 60s	1229 ± 4.76	1.22
CBZ-PVP 40-60 (20%ASD) 150MPa 30s	1202 ± 0.88	1.19
CBZ-PVP 40-60 (20%ASD) 250MPa 5s	1138 ± 1.52	1.13
CBZ-PVP 40-60 (20% ASD) 250MPa 60s	1095 ± 3.09	1.09
CBZ-PVP 20-80 (50%ASD) 50MPa 5s	1204 ± 2.00	1.19
CBZ-PVP 20-80 (50%ASD) 50MPa 60s	1193 ± 3.25	1.18
CBZ-PVP 20-80 (50%ASD) 150MPa 30s	1128 ± 3.03	1.12
CBZ-PVP 20-80 (50%ASD) 250MPa 5s	1071 ± 3.65	1.06
CBZ-PVP 20-80 (50% ASD) 250MPa 60s	1038 ± 1.70	1.03
CBZ crystalline	1008 ± 1.0	1.00

<u>Figure S1</u>: **(a)** DSC thermograms; **(b)** ATR-FTIR spectra and **(c)** pXRD diffractograms of IND ASDs and pure materials.

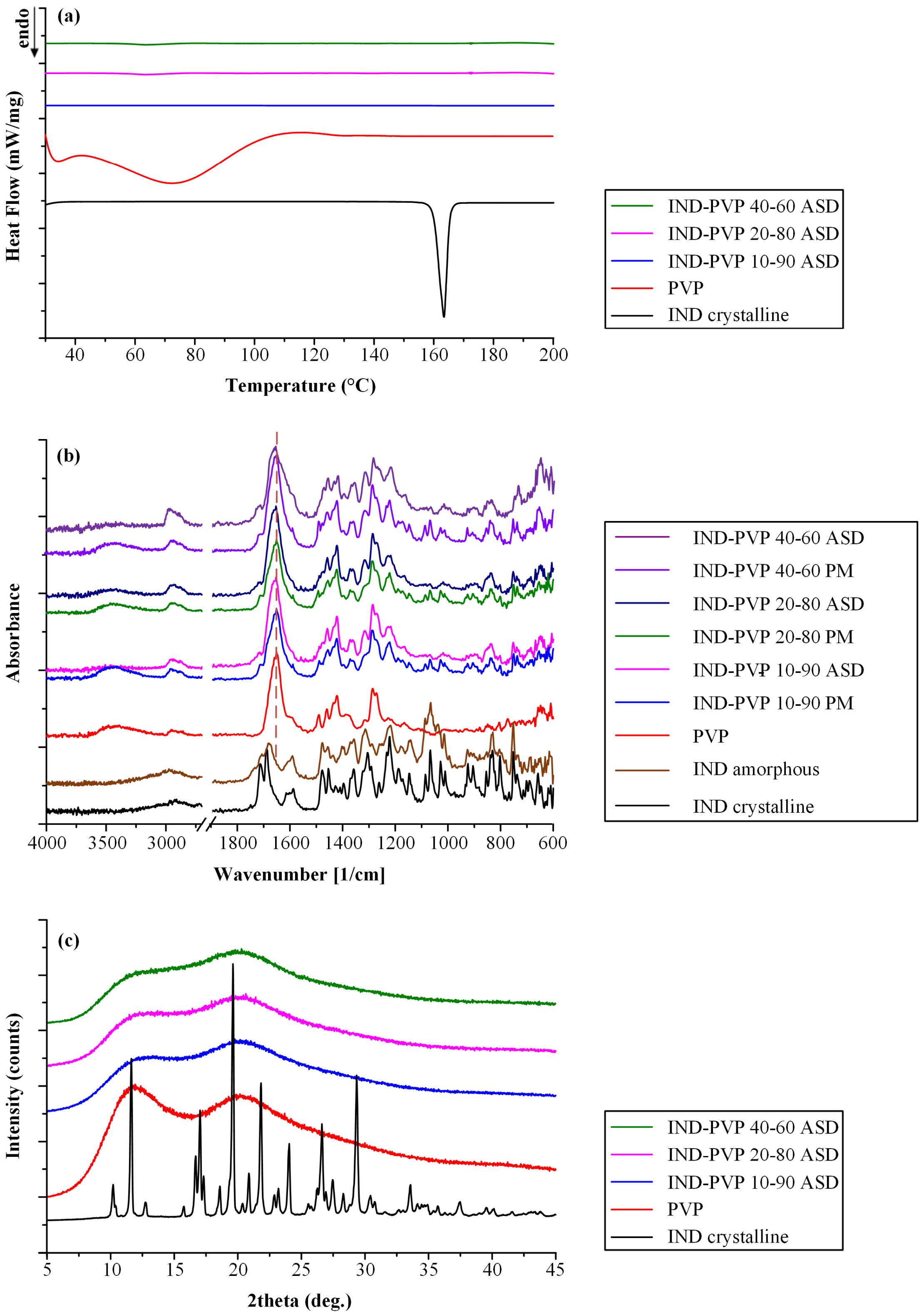

Figure S2: **(a)** DSC thermograms; **(b)** ATR-FTIR spectra pXRD diffractograms and **(c)** pXRD diffractograms of CBZ ASDs and pure materials.

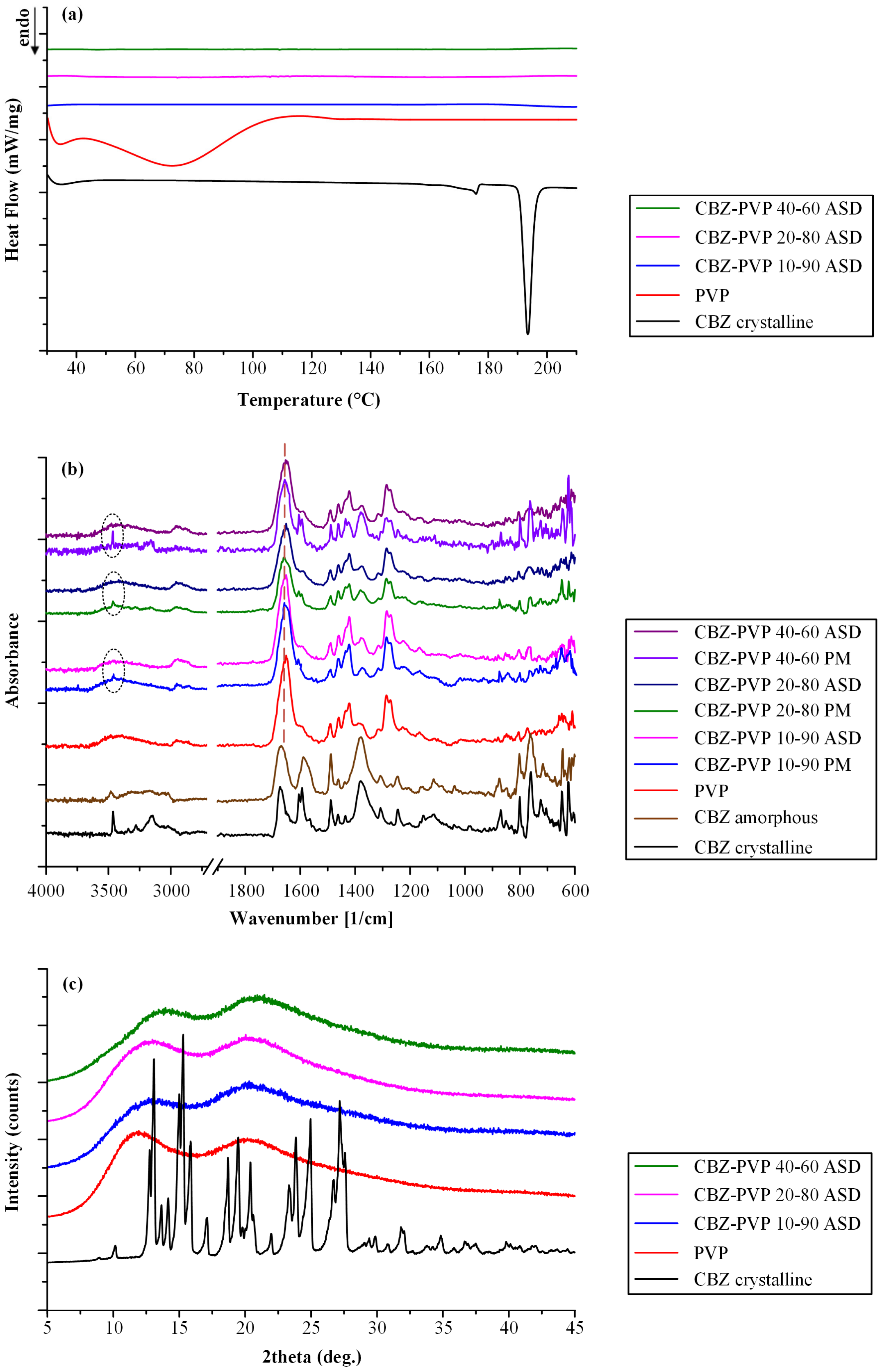

<u>Figure S3</u>: XRD diffractograms of IND-PVP tablets after 3 months of storage (25^{0}C, 60 % RH).

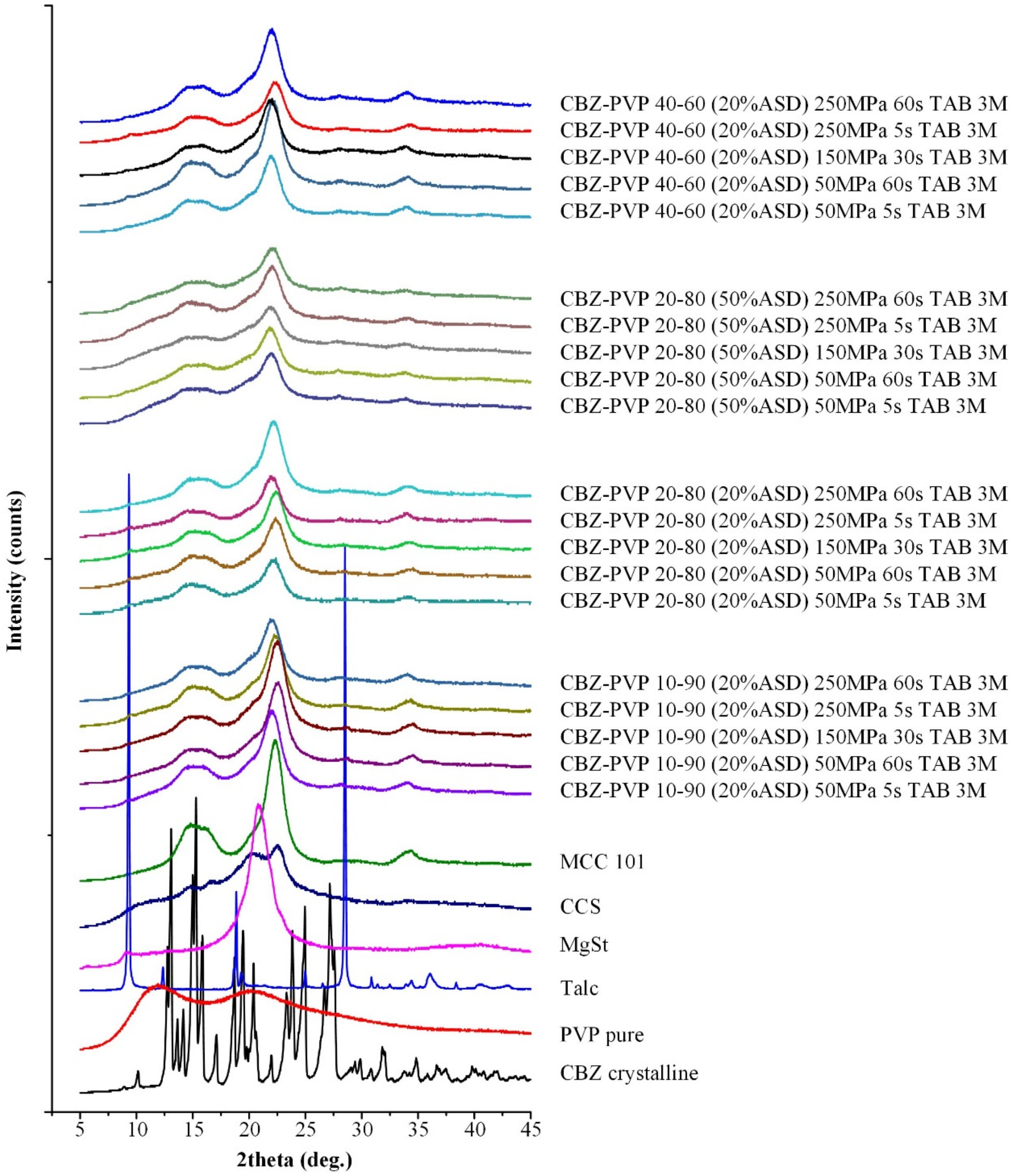

<u>Figure S4</u>: XRD diffractograms of IND-PVP tablets after 3 months of storage (25^{0}C, 60 % RH).

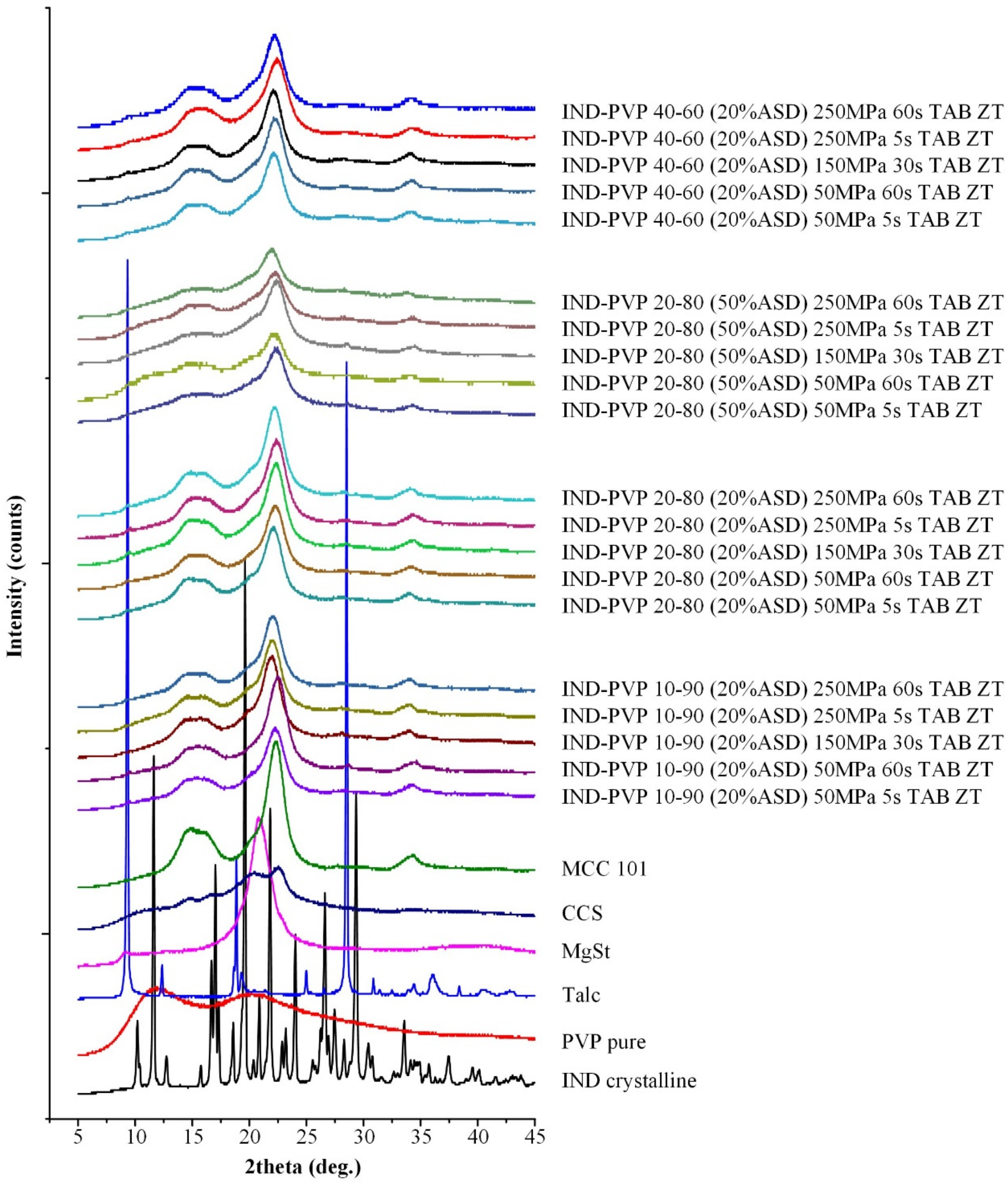

Supplement: Supplementary file 1 [file polymers-17-02484-s001.zip › polymers-3829762-supplementary.pdf]
